# Co‐development of ADHD symptoms and emotional problems from childhood to adulthood: predictors and developmental outcomes

**DOI:** 10.1111/jcpp.70196

**Published:** 2026-06-30

**Authors:** Yuan You, Helena M.S. Zavos, Laurie J. Hannigan, Linlin Zhang, Giacomo Bignardi, Meredith X. Han, Elisavet Palaiologou, Christopher Rayner, Tom A. McAdams

**Affiliations:** ^1^ Social, Genetic and Developmental Psychiatry Centre Institute of Psychiatry, Psychology & Neuroscience, King's College London London UK; ^2^ Department of Psychology, Institute of Psychiatry, Psychology & Neuroscience King's College London London UK; ^3^ Research Department Lovisenberg Diaconal Hospital Oslo Norway; ^4^ Centre for Genetic Epidemiology and Mental Health Norwegian Institute of Public Health Oslo Norway; ^5^ Population Health Sciences Bristol Medical School, University of Bristol Bristol UK; ^6^ School of Psychology, Capital Normal University Beijing China; ^7^ Department of Paediatrics Yong Loo Lin School of Medicine, National University of Singapore Singapore City Singapore

**Keywords:** ADHD, emotional problems, joint‐trajectory analysis, early‐life predictors, developmental outcomes

## Abstract

**Background:**

Attention‐deficit/hyperactivity disorder (ADHD) symptoms and emotional problems frequently co‐occur. Longitudinal data provide opportunities to understand how and why they co‐develop.

**Methods:**

Data were drawn from the Twins Early Development Study, which included 27,890 participants. Emotional problems and ADHD symptoms were parent‐reported using the Strengths and Difficulties Questionnaire and Conners' Parent Rating scales. We modeled the co‐development of ADHD symptoms and emotional problems from ages 4 to 21 using joint trajectory analysis. The predictors included polygenic scores (PGSs) for multiple psychiatric symptoms, maternal depression, family chaos, and socioeconomic status (SES). The outcomes included SES, lifetime psychiatric diagnoses, undergraduate diploma attainment, and entry into parenthood at age 26.

**Results:**

A four‐class joint trajectory model was selected as best fitting and included: ‘Low ADHD, Low emotional problems’, ‘High‐decreasing ADHD, Low emotional problems’, ‘Mid‐decreasing ADHD, Increasing emotional problems’, and ‘Increasing ADHD, Increasing emotional problems’. The consistently low‐symptom group reported lower ADHD and had lower externalizing PGSs, lower maternal depression, higher SES, and less home chaos at baseline, and higher educational attainment and fewer lifetime depression/anxiety diagnoses at 26 than groups with higher symptom levels. Those with high ADHD symptoms in childhood or increasing ADHD symptoms from childhood to adulthood had the lowest educational attainment and SES in adulthood.

**Conclusions:**

Groups characterized by increasing ADHD symptoms also tended to show increases in emotional problems, whereas increases in emotional problems did not necessarily coincide with increases in ADHD symptoms. The trajectories had distinct early‐life predictors and adult outcomes, highlighting potential intervention targets.

## Introduction

ADHD symptoms and emotional problems not only co‐occur but are also associated with the persistence and emergence of each other. In comparison to children diagnosed with only ADHD, those with ADHD and comorbid anxiety or depression are more likely to experience persistent ADHD symptoms into adulthood (You et al., 2024) and struggle with academic performance in later stages of education (Cuffe et al., 2020).

From a developmental perspective, ADHD and emotional problems may increase, remain stable, or decrease across developmental stages (Murray et al., 2022; Tandon, Tillman, Agrawal, & Luby, 2016). Some individuals exhibit both symptoms, whereas others show elevations in only one of the two (e.g. Biederman, Petty, Clarke, Lomedico, & Faraone, 2011; D'Agati, Curatolo, & Mazzone, 2019). The co‐development of externalizing and emotional problems has been studied. For example, Hannigan et al. (2018) tracked conduct and emotional problems from ages 4 to 16. Most participants (54.2%) belonged to a ‘normative’ group with consistently low symptoms, while others were in ‘Low/decreasing conduct and emotional problems’ (23.8%), ‘Emotional problems only’ (14.7%), or ‘Elevated/increasing symptoms’ (7.3%) groups. PGSs for years of education and depressive symptoms were associated with membership in the elevated/increasing symptom group. In another study of the joint trajectories of internalizing and externalizing behaviors from age 4 to 16 years old, five classes were identified (Speyer et al., 2022). Lower maternal age, maternal smoking, maternal psychiatric problems, and child PGSs for ADHD and schizophrenia were found to distinguish between classes. However, while previous studies have examined the co‐development of broader externalizing problems (which may include ADHD symptoms) and emotional problems, none have specifically investigated the co‐development of ADHD and emotional problems. As a result, it remains unclear how ADHD and emotional problems co‐develop over time and whether ADHD subdomains, inattention and hyperactivity‐impulsivity, follow distinct co‐development trajectories with emotional problems. Specifically, in comparison to the hyperactivity‐impulsivity subdomain, inattention tends to remain stable across development (Larsson, Dilshad, Lichtenstein, & Barker, 2011; Pingault et al., 2011) and becomes more strongly associated with emotional problems (You et al., 2024). Differences in the developmental trajectories of ADHD subdomains may also manifest in different co‐developmental patterns with emotional problems, which warrants further investigation.

The overlap between ADHD symptoms and emotional problems is partly attributable to shared genetic factors (Andersson et al., 2020; Du Rietz et al., 2018; Powell, Martin, Thapar, Rice, & Anney, 2021; Schmitz & Mrazek, 2001). PGSs for ADHD and depression each predict the long‐term development of both ADHD and emotional problems (e.g. Avinun & Hariri, 2019; Grimes et al., 2024; Kwong et al., 2021; Mitchell et al., 2021; Riglin et al., 2016; Schultebraucks, Choi, Galatzer‐Levy, & Bonanno, 2021). Moreover, depression PGS (Benca et al., 2017; Schork et al., 2019) is closely linked to executive functioning, and anxiety PGS has been found to show a negative association with total IQ and processing speed index (Wu et al., 2025). These executive functions, in turn, are associated with the development and persistence of ADHD symptoms (Roselló et al., 2020). ADHD PGS is also associated with emotion dysregulation (Nigg et al., 2020), a key factor in depression onset (Kovacs, Joormann, & Gotlib, 2008). The shared functional impairments predicted by emotional problems and ADHD PGSs suggest they may be associated with the co‐development of these symptoms. In addition to psychiatric disorder PGSs, educational attainment PGS is associated with inhibitory control (Rea‐Sandin et al., 2021) and cognitive and non‐cognitive skills (Judd et al., 2020), which may also influence both emotional problems and ADHD symptoms (Bäckman et al., 2024; Piazza et al., 2024) in the short and long term.

Beyond genetic influences, family environment plays an important role in development (Bronfenbrenner, 2005). For example, family cohesion and maternal depression have been shown to play a significant role in the comorbidity of ADHD and emotional problems (Borden et al., 2020). These early environmental exposures may influence the development of mental health symptoms in children through parenting behaviors, family stress, and differences in resource investment (Goodman, 2020; Miller & Votruba‐Drzal, 2017).

Previous research has also explored how individuals with different developmental patterns of ADHD and emotional problems differ in terms of mental health and other developmental outcomes. Compared to groups with consistently low levels of ADHD or emotional problems, individuals with higher or persistent symptoms report more mental health problems, higher rates of school dropout and unemployment (Carter, Speyer, Caye, Rohde, & Murray, 2025; Musliner, Munk‐Olsen, Eaton, & Zandi, 2016; Sasser, Kalvin, & Bierman, 2016). In addition, the co‐occurrence of ADHD with other psychiatric conditions has been associated with worse outcomes than ADHD alone. Children with ADHD and comorbid emotional problems often experience reduced quality of life and more mental health challenges (Armstrong, Lycett, Hiscock, Care, & Sciberras, 2015; Uchida, Spencer, Faraone, & Biederman, 2018), as well as adverse educational and occupational outcomes (Jangmo et al., 2021; Nelson & Gregg, 2012). In addition, from a life course perspective, entry into parenthood is an important transitional experience in adulthood (e.g. Nomaguchi & Milkie, 2003; Umberson, Pudrovska, & Reczek, 2010). In previous studies, both depression and persistent ADHD symptoms have been linked to an increased likelihood of early pregnancies (Maravilla, 2019; Meinzer et al., 2020).

In the current study, we identified joint developmental trajectories of ADHD symptoms and emotional problems, and the predictors and outcomes associated with these trajectories. We expected that ADHD symptoms and emotional problems would co‐develop for some people but be independent for others (Brown, 2009). Therefore, we anticipated four joint developmental trajectories: high ADHD symptoms with high emotional problems, low ADHD symptoms with low emotional problems, high ADHD with low emotional problems, and low ADHD symptoms with high emotional problems.

PGSs for anxiety, depression, ADHD, and educational attainment, and family environment factors (e.g. childhood socioeconomic status, family chaos) were incorporated as possible predictors of class membership. Mental health outcomes and indices of SES were included as adult outcomes. We anticipated that participants would differ in psychiatric PGSs and early environmental exposures across trajectory classes, and that some joint developmental trajectories would be associated with lifetime psychiatric diagnosis by 26 years old, educational attainment, entry into parenthood, and SES. We conducted exploratory analyses to explore whether patterns of results differed between ADHD subdomains.

## Methods

Analyses were pre‐registered on the Open Science Framework (OSF) website before accessing the data: https://osf.io/cjdzk.Our analyses closely aligned with the pre‐registration on OSF. The only variation was the addition of ‘entry into parenthood’ as an outcome variable.

### Participants

We used data from the Twins Early Development Study (TEDS). TEDS is an ongoing longitudinal cohort study focused on the cognitive, behavioral, and emotional development of over 13,000 twin pairs from childhood through adulthood (Lockhart et al., 2023). The analyses presented in this article included 25,876 participants. Written informed consent was obtained from parents and twins.

### Measures

#### Emotional problems

Emotional problems were reported by parents using the strengths and difficulties questionnaire (SDQ7; Goodman, 1997) at ages 4, 7, 9, 12, and 21. The SDQ emotional symptoms subscale consists of 5 items. All items were reported on a 3‐point Likert scale. One example item is ‘Many worries, often seems worried’. Total scores at each wave were used in trajectory models, with at least three non‐missing items required to compute the scale.

#### *ADHD* *symptoms*

ADHD symptoms were assessed using 18 items from the Conners parent rating scale ADHD symptom subscale (Conners et al., 1997) when twins were 8, 12, 14, 16, and 21 years old. All items are reported on a 4‐point scale. One example item is ‘talks excessively’. Of the 18 items, 9 measured hyperactivity‐impulsivity and 9 measured inattention. Item scores were summed at each wave. To construct the scale, at least nine items were required to be non‐missing.

#### Predictors associated with the trajectories

##### PGSs for psychopathologies and educational attainment

We used PGSs capturing ADHD (Demontis et al., 2023), depression (Howard et al., 2019), anxiety (Purves et al., 2020), MDD (Wray et al., 2018), and educational attainment (Okbay et al., 2022). In the total sample, PGS data were available for 13,016 participants. PGS were created using the LDpred software (Vilhjálmsson et al., 2015). Details regarding the creation of these PGS can be found in the TEDS data dictionary: https://www.teds.ac.uk/datadictionary/studies/measures/polygenic_scores.htm.

##### Family environment factors

Family SES was indexed via a composite score derived from parental educational level and employment status when the TEDS twins were 1.5 years old (Hanscombe et al., 2012).

Maternal depression was assessed with the 10‐item self‐report Edinburgh Postnatal Depression Scale (EPDS; Cox, Holden, & Sagovsky, 1987) reported by mothers when children were 3 and 4 years old. Responses are rated on a 4‐point Likert scale. An example item is ‘I have felt happy’.

Chaos at home was measured with the 15‐item Confusion, Hubbub and Order Scale (CHAOS; Matheny Jr, Wachs, Ludwig, & Phillips, 1995) when children were 3 years old. It is a 4‐point Likert scale reported by parents. An example item is ‘There is very little commotion in our home’.

#### Predicting adult outcomes from joint trajectories

##### SES

Adult SES was collected when the twins were 26 years old and was a composite of three standardized indicators: educational level, income level, and economic vulnerability. Educational level was assessed using an 11‐level categorical variable ranging from ‘none’ to ‘PhD’. The economic vulnerability score is a sum of five 1/0 flags including the following variables: not in education, employment or training; low income; having children; on benefits; zero‐hours employment. The employment categories refer to the Standard Occupational Classification (SOC, 2000).

##### Educational attainment

The highest level of educational attainment was reported at age 26 using a 10‐point self‐report scale, ranging from ‘No qualifications’ to ‘Doctoral degree’.

##### Entry into parenthood

A binary variable indicating entry into parenthood (0 = no child, 1 = has child) was created using parent‐ and twin‐reports at ages 21 and 26. TEDS participants with a child enrolled in the Children of Twins Early Development Study (CoTEDS; Ahmadzadeh et al., 2019; Lockhart et al., 2023) were identified as a parent. Positive pregnancy reports from the Coddington Life Events Scale (CLES; Coddington, 1999) were also used to identify parents, excluding participants who experienced miscarriage or abortion. Those who reported no child or pregnancy at wave 21 or wave 26 were classified as without a child.

##### Lifetime mental health diagnoses

Lifetime mental health diagnoses, including depression, anxiety, personality disorder, and ADHD, were assessed at age 26 using the General Mental Health (GMH) measure. This consisted of two multipart questions developed by researchers from the Genetic Links to Anxiety and Depression (GLAD; Davies et al., 2019) study, asking: ‘Have you ever been diagnosed with one or more of the following mental health problems by a professional, even if you do not currently have it?’

#### Validation variables

Several self‐reported measures of mental health symptoms at 26 years old were included for validation of the joint trajectories: SDQ emotional symptoms, SDQ hyperactivity (Goodman, 1997) and Conners inattention (Conners et al., 1997). SDQ emotional symptoms and SDQ hyperactivity each consist of five items. The Conners inattention scale consists of 11 items.

### Data analysis

Mplus 8.3 and R 4.2.1 were used to analyze the data. To account for the non‐independence of twins, participants were clustered based on family ID. In Mplus, the ‘TYPE = COMPLEX MIXTURE’ allows for the estimation of latent growth models with clustered data. Missing data in this study were handled using full information maximum likelihood in Mplus.

#### Baseline models

First, we employed single‐phenotype latent growth models to capture the developmental trajectories of ADHD symptoms, inattention, hyperactivity‐impulsivity, and emotional problems from early childhood to adulthood.

#### Parallel Process Latent Class Growth Analysis

Parallel Process Latent Class Growth Analysis (PP‐LCGA) is a latent class analysis method that integrates the developmental trajectories of multiple traits by modeling their co‐development (Hannigan et al., 2018; Speyer et al., 2022). This approach was used to identify the co‐development of ADHD symptoms and emotional problems in the current study. The intercept growth factor was allowed to vary across classes for different class solutions (ranging from 2 to 6 classes). Three joint trajectory models were examined: ADHD and emotional problems; inattention and emotional problems; and hyperactivity and emotional problems.

Multiple models were fitted to the data, each with a different number of co‐development classes. Akaike information criterion (AIC; Akaike, 1987), Bayesian Information Criterion (BIC; Schwarz, 1978), adjusted Bayesian information criterion (aBIC; Sclove, 1987), entropy (Muthen & Muthen, 2010), and Lo–Mendel–Rubin test (LMRT; Vermunt, 2024) were used to choose the best‐fitting models. Generally, a smaller AIC and BIC indicate better model fit. LMRT provided a test of whether a model with more classes fit better than a model with less (*p* < .05). In addition, entropy reflects the reliability of the model. An entropy score higher than 0.6 indicates that the reliability of the classes in latent growth models is acceptable. In this study, we primarily used LMRT and entropy to determine the optimal number of classes. We interpret classes comprising at least 2% of the sample, consistent with prior ADHD trajectory studies reporting classes of 2%–3% (Döpfner et al., 2015; Riglin et al., 2016).

The means and standard deviations of the predictor and outcome variables within each class were calculated based on the most likely latent class membership. In the subsequent pairwise comparisons of predictors and outcomes across different classes, in order to account for the uncertainty in joint trajectory classification, auxiliary variables, including predictor and outcome variables, were used when testing for associations between class membership, predictors (ML three‐step approach; Vermunt, 2010), and outcomes (Bolck, Croon, & Hagenaars, 2004; Lanza, Tan, & Bray, 2013). Specifically, we adjusted for baseline SES in tests of the associations between class membership and outcomes. By using auxiliary variables, classification error is incorporated, meaning that individuals are assigned to latent categories with a certain probability (Nylund‐Gibson et al., 2019).

In order to account for multiple testing, we performed a Bonferroni correction. Specifically, because we test for associations between class membership and 16 risk‐factors/outcome variables, we consider a variable to be associated with the grouping of joint developmental trajectories when *p* < .05/16 ≈ 0.0031.

To account for potential bias from nonrandom sample attrition, we used early baseline variables available in TEDS to construct inverse probability weights (IPW; Seaman & White, 2013) based on whether participants had at least two time points of both ADHD and emotional problems assessments across all five waves. In addition, multiple imputation (MI; Sinharay, Stern, & Russell, 2001) was applied to address missing data in trajectory predictors and outcomes.

## Results

### Descriptive statistics and correlations

Descriptive statistics are shown in Table S1. The sex distribution in our sample was relatively balanced, with females accounting for 50.1%. The majority of participants were White (91.9%).

Correlations between ADHD and emotional problems are shown in Table 1. Measures of ADHD and emotional problems correlated with each other within and across timepoints (*r* = .12 to .32). Correlations increased as measurement ages became closer. The lowest correlation was between ADHD symptoms at age 21 and emotional problems at age 4, while the highest correlation was observed between ADHD symptoms at age 21 and emotional problems at age 21. On average, ADHD symptoms showed a declining trend with age from childhood (*M* = 11.17) to adulthood (*M* = 6.48). To check whether this decline represented a developmental trend or was the result of selective attrition over time, we compared participants with complete data to those who dropped out at any waves. Results indicated that after adjusting for within‐pairs correlation, having complete trajectory data was not associated with baseline emotional problems (*p* = .10) or ADHD symptoms (*p* = .08). Correlations between ADHD subdomains and emotional problems are shown in Appendix S1 and Table S2.

**Table 1:** The correlation of ADHD and emotional problems measured at different time points

Symptoms	*M* (*SD*)	1	2	3	4	5	6	7	8	9	10
1 Age 4 EP	1.39 (1.44)	1									
2 Age 7 EP	2.20 (1.84)	.38***	1								
3 Age 9 EP	1.73 (1.91)	.36***	.51***	1							
4 Age 12 EP	1.82 (1.93)	.31***	.45***	.52***	1						
5 Age 21 EP	2.37 (2.29)	.19***	.30***	.37***	.39***	1					
6 Age 8 ADHD	11.17 (9.35)	.16***	.21***	.24***	.23***	.15***	1				
7 Age 12 ADHD	9.85 (8.61)	.16***	.17***	.25***	.31***	.16***	.70***	1			
8 Age 14 ADHD	8.66 (8.43)	.14***	.15***	.19***	.22***	.16***	.60***	.74***	1		
9 Age 16 ADHD	6.81 (7.41)	.14***	.16***	.21***	.23***	.21***	.52***	.64***	.73***	1	
10 Age 21 ADHD	6.48 (7.11)	.12***	.14***	.18***	.19***	.32***	.42***	.50***	.56***	.58***	1

ADHD, attention‐deficit/hyperactivity disorder; EP, emotional problems.

*p < .05, **p < .01, ***p < .001.

### Single‐phenotype latent growth models

For details on the single‐phenotype latent growth modeling analysis, see Appendix S2, Tables S3–S6 and Figure S1.

### Joint trajectories of ADHD and emotional problems

A 4‐class model achieved the best fit for the joint ADHD and emotional problems (see Table 2 and Figure 1). This included the following categories: ‘Low ADHD, Low emotional problems’, ‘High‐decreasing ADHD, Low emotional problems’, ‘Mid‐decreasing ADHD, Increasing emotional problems’ and ‘Increasing ADHD, Increasing emotional problems’. Groups characterized by increasing ADHD symptoms also tended to show increases in emotional problems, whereas increases in emotional problems did not necessarily coincide with increases in ADHD symptoms.

**Table 2:** Relative fit indices for 1 through 5 class models for joint trajectories of ADHD and emotional problems

Number of classes	AIC	BIC	ABIC	LMRT	Entropy
1	569,846	569,981	569,926	–	–
2	564,700	564,874	564,804	0.000	0.828
3	561,530	561,743	561,658	0.000	0.802
**4**	**559,777**	**560,030**	**559,928**	**0.000**	**0.779**
5	558,394	558,687	558,569	0.207	0.768

Bold values indicate that the 4‐class model provides the best fit to the data.

ABIC, adjusted Bayesian information criterion; AIC, Akaike information criterion; BIC, Bayesian information criterion; LMRT, Lo–Mendel–Rubin test.

**Figure 1:**
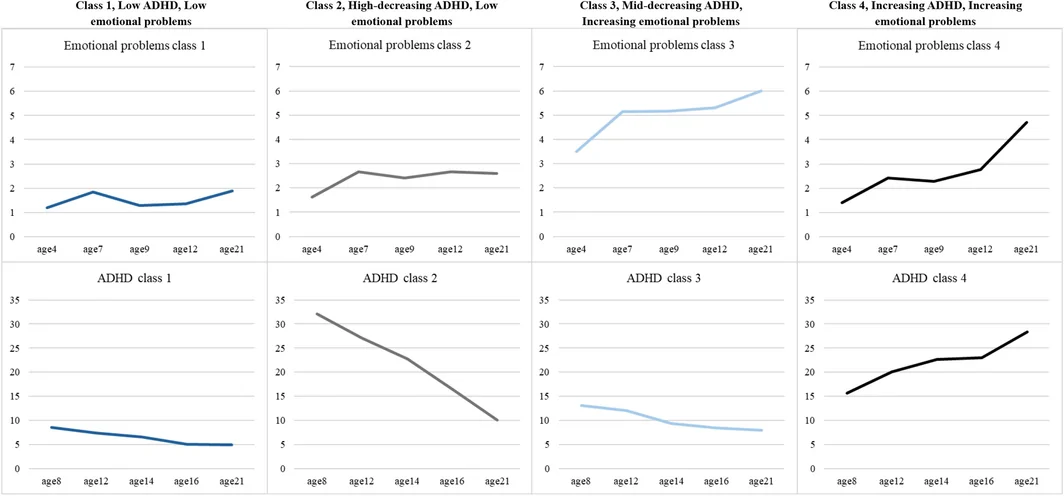
Joint trajectories of ADHD and emotional problems. Class 1, Low ADHD, Low emotional problems (*N* = 16,338, 81.4%, SlopeADHD = −0.28, InterceptADHD = 8.37, SlopeEP = 0.03, InterceptEP = 1.29); class 2: High‐decreasing ADHD, Low emotional problems (*N* = 1,457, 7.3%, S_ADHD_ = −1.70, I_ADHD_ = 32.32, S_EP_ = 0.07, I_EP_ = 2.00); class 3: Mid‐decreasing ADHD, Increasing emotional problems (*N* = 1,580, 7.9%, S_ADHD_ = −0.37, I_ADHD_ = 12.68, S_EP_ = 0.15, I_EP_ = 3.71); class 4: Increasing ADHD, Increasing emotional problems (*N* = 695, 3.5%, S_ADHD_ = 0.99, I_ADHD_ = 15.79, S_EP_ = 0.19, I_EP_ = 1.48). Sample size: 20074. ADHD, attention‐deficit/hyperactivity disorder; EP, emotional problems

Trajectory classes were validated using measures of inattention, hyperactivity, and emotional problems at age 26 (see Tables S7 and S8). Results supported the validity of the trajectory classification (see Appendix S3).

#### Predictors associated with the trajectories

We explored associations between PGSs and joint trajectory class membership, as shown in Tables 3 and 4 and Figure 2. Participants in the ‘High‐decreasing ADHD, Low emotional problems’ group had a significantly higher ADHD PGS (*M* = 0.28) than participants in the ‘Low emotional problems, Low ADHD’ group (*M* = −0.05). Participants in the ‘Mid‐decreasing ADHD, Increasing emotional problems’ group exhibited significantly lower externalizing PGS (*M* = −0.06) compared to the ‘High‐decreasing ADHD, Low emotional problems’ (*M* = 0.22) group and the ‘Increasing ADHD, Increasing emotional problems’ group (*M* = 0.34). There were no associations between anxiety PGS, depression PGS, MDD PGS, or educational attainment PGS and joint trajectory class membership.

**Table 3:** Descriptive statistics of predictor and outcome variables for the joint trajectory of ADHD and emotional problems based on most likely latent class membership

Trajectory group (*M*, *SD*/*N*, %)						
		*n*	1. Low ADHD, low EP (81.4%)	2. High‐decreasing ADHD, low EP (7.3%)	3. Mid‐decreasing ADHD, increasing EP (7.9%)	4. Increasing ADHD, increasing EP (3.5%)
Predictors (*M*, *SD*)	ADHD PGS	12,543	−0.05 (1.00)	0.28 (0.97)	0.13 (1.01)	0.26 (0.99)
	Anxiety PGS	12,543	0.02 (1.00)	−0.05 (1.04)	−0.13 (1.01)	−0.08 (0.97)
	Depression PGS	12,543	−0.04 (1.00)	0.10 (0.99)	0.19 (1.00)	0.21 (1.02)
	EA PGS	12,543	0.04 (1.00)	−0.18 (0.99)	−0.16 (0.94)	−0.13 (1.02)
	Externalizing PGS	12,543	−0.03 (1.00)	0.22 (0.98)	−0.06 (1.00)	0.34 (1.01)
	MDD PGS	12,543	−0.03 (1.00)	0.07 (0.98)	0.13 (1.00)	0.18 (1.01)
	Maternal depression	10,692	−0.09 (0.96)	0.37 (1.05)	0.51 (1.06)	0.27 (1.08)
	Family chaos	10,676	−0.09 (0.98)	0.42 (0.97)	0.44 (1.00)	0.31 (0.95)
	SES	18,769	0.04 (1.00)	−0.24 (0.97)	−0.16 (1.00)	−0.08 (1.05)
Continuous outcomes (*M*, *SD*)	SES	7,999	0.09 (0.94)	−0.43 (1.16)	−0.33 (1.13)	−0.58 (1.26)
	Depression diagnosis	7,790	1,433 (22.3%)	148 (32.4%)	308 (46.8%)	128 (49.8%)
	Anxiety diagnosis	7,790	614 (9.6%)	47 (10.3%)	135 (20.5%)	45 (17.5%)
	PD diagnosis	7,783	66 (1.0%)	7 (1.5%)	19 (2.9%)	13 (5.2%)
Categorical outcomes (*N*, %)	ADHD diagnosis	7,783	71 (1.1%)	25 (5.5%)	16 (2.4%)	23 (9.2%)
	Undergraduate diploma	7,845	4,529 (70.0%)	197 (43.4%)	380 (56.5%)	116 (46.0%)
	Entry into parenthood	8,347	946 (13.9%)	122 (23.9%)	128 (17.8%)	83 (27.8%)

All predictors and outcomes have been standardized to have a mean of zero and *SD* of 1. Trajectory group (*M*, *SD*): The mean and standard deviation of predictors in each trajectory group based on the most likely latent class membership. Trajectory group (*N*, %): The number and proportion of participants in each trajectory group based on the most likely latent class membership. ADHD, attention‐deficit/hyperactivity disorder; EA, educational attainment; EP, emotional problems; *M*, mean; MDD, major depressive disorder; PD, personality disorder; PGS, polygenic risk scores; OR, odds ratio; *SD*, standard deviation; SES, socioeconomic status.

**Table 4:** Pairwise comparisons of predictor and outcome variables for the joint trajectory of ADHD and emotional problems across classes in the three‐step method

Group comparison (OR, *SE*/Chi‐square)							
		2 vs. 1	3 vs. 1	4 vs. 1	3 vs. 2	4 vs. 2	4 vs. 3
Predictors (OR, *SE*)	**ADHD PGS**	1.44 (0.12)*	1.35 (0.12)	1.30 (0.14)	0.94 (0.10)	0.90 (0.12)	0.96 (0.13)
	Anxiety PGS	1.04 (0.09)	0.91 (0.08)	1.13 (0.12)	0.87 (0.10)	1.09 (0.16)	1.23 (0.17)
	Depression PGS	1.02 (0.10)	1.08 (0.10)	1.27 (0.17)	1.06 (0.14)	1.25 (0.21)	1.18 (0.18)
	EA PGS	0.91 (0.09)	0.82 (0.08)	1.03 (0.11)	0.90 (0.11)	1.13 (0.16)	1.26 (0.16)
	**Externalizing PGS**	1.10 (0.09)	0.82 (0.07)	1.38 (0.14)	0.75 (0.08)*	1.25 (0.16)	1.68 (0.21)*
	MDD PGS	0.95 (0.09)	1.02 (0.09)	1.09 (0.13)	1.07 (0.13)	1.15 (0.17)	1.08 (0.15)
	**Maternal depression**	1.51 (0.13)*	1.79 (0.13)*	1.36 (0.15)	1.19 (0.12)	0.90 (0.13)	0.76 (0.10)
	**Family chaos**	1.53 (0.14)*	1.67 (0.16)*	1.54 (0.17)*	1.09 (0.12)	1.01 (0.13)	0.92 (0.12)
	**SES**	0.77 (0.07)*	0.87 (0.09)	0.93 (0.10)	1.13 (0.14)	1.20 (0.17)	1.06 (0.15)
Continuous outcomes (Chi‐Square)	**SES**	35.61*	50.28*	49.04*	0.04	7.73	7.97
Categorical outcomes (OR, *SE*)	**Depression diagnosis**	1.58 (0.24)*	4.42 (0.56)*	5.32 (1.00)*	2.80 (0.51)*	3.36 (0.81)*	1.20 (0.26)*
	**Anxiety diagnosis**	1.09 (0.25)*	3.33 (0.48)*	2.42 (0.56)*	3.07 (0.77)*	2.23 (0.72)*	0.73 (0.18)*
	PD diagnosis	0.98 (0.84)	3.48 (1.45)	8.09 (3.29)	3.53 (3.14)	8.20 (7.68)	2.32 (1.09)
	ADHD diagnosis	8.32 (3.18)	4.05 (1.73)	17.67 (6.62)	0.49 (0.21)	2.12 (0.85)	4.37 (1.85)
	**Undergraduate diploma**	0.29 (0.04)*	0.54 (0.08)*	0.25 (0.06)*	1.86 (0.36)*	0.87 (0.24)*	0.47 (0.12)*
	**Entry into parenthood**	1.51 (0.25)*	1.21 (0.19)*	2.52 (0.54)*	0.80 (0.17)*	1.67 (0.44)*	2.09 (0.53)*

1. Low ADHD, low EP (81.4%); 2. High‐decreasing ADHD, low EP (7.3%); 3. Mid‐decreasing ADHD, increasing EP (7.9%); 4. Increasing ADHD, increasing EP (3.5%). Baseline SES was controlled for when examining the associations between trajectory classes and outcomes. An odds ratio (OR) greater than 1.00 in the 2 vs. 1 comparison column was associated with higher odds of class membership in group 2 (comparison group) compared to group 1 (reference group). For example, a higher PGS for ADHD was associated with greater odds of class membership in group 2 compared to group 1. Bold text indicates significant predictor and outcome variables.

* After Bonferroni correction, the *p*‐value reached the significance level.

**Figure 2:**
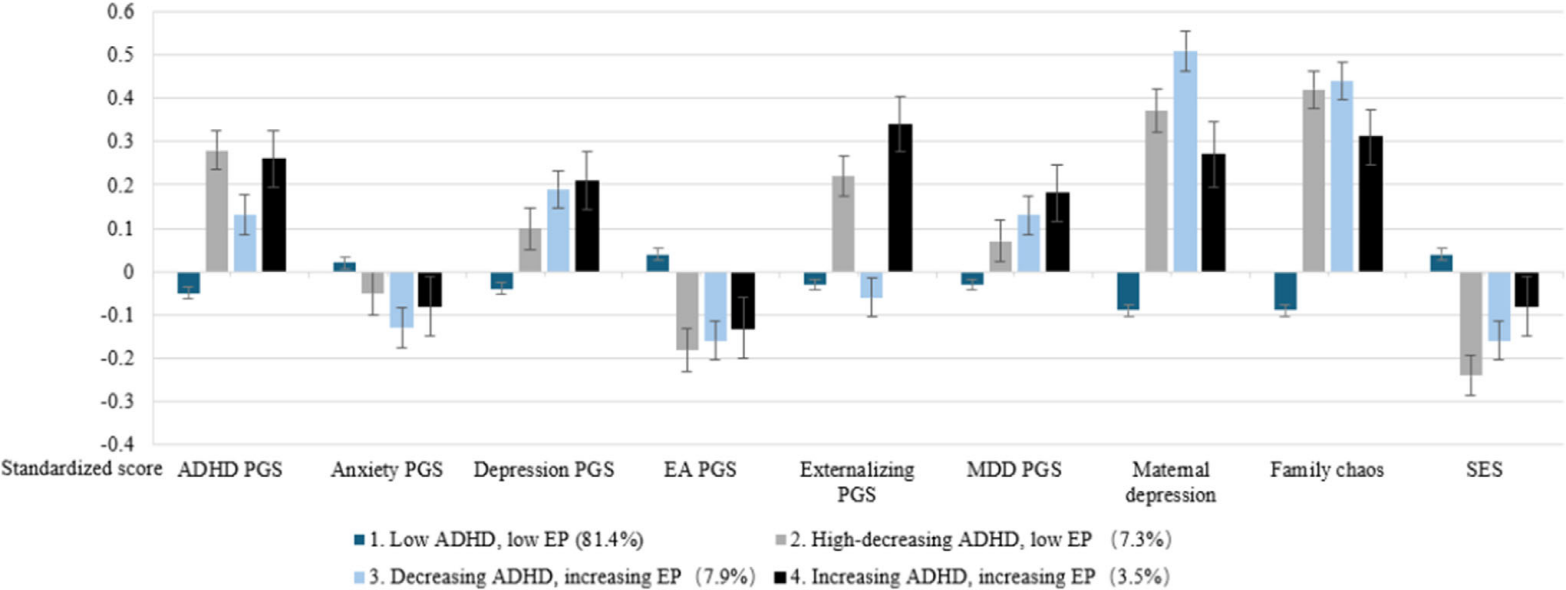
Predictors for the joint trajectory of ADHD and emotional problems based on most likely latent class membership. All predictors have been standardized to have a mean of zero and *SD* of 1. The error bars represent the standard error of the data. ADHD, attention‐deficit/hyperactivity disorder; EA, educational attainment; EP, emotional problems; MDD, major depressive disorder; PD, personality disorder; PGS, polygenic risk scores; SES, socioeconomic status

We also explored associations with indices of the childhood environment. Participants in the ‘High‐decreasing ADHD, Low emotional problems’ group and the ‘Mid‐decreasing ADHD, Increasing emotional problems’ group had mothers with higher levels of depression (*M* = 0.37 and 0.51) than those in the ‘Low ADHD, Low emotional problems’ group (*M* = −0.09). Participants in all higher symptom groups reported greater family chaos (*M* = 0.31 to 0.44) compared to the ‘Low ADHD, Low emotional problems’ group (*M* = −0.09). SES was lower in the ‘High‐decreasing ADHD, Low emotional problems’ group (*M* = −0.24) compared to the ‘Low ADHD, Low emotional problems’ group (*M* = 0.04). Overall, participants in the early low‐symptom groups tended to come from more favorable family environments, characterized by low maternal depression, low family chaos, and high SES.

#### Predicting adult outcomes from joint trajectories

As for the mental health outcomes of the ADHD–emotional problems trajectories (see Tables 3 and 4 and Figure 3), participants in the ‘Low ADHD, Low emotional problems’ group had lower rates of depression diagnoses and anxiety diagnoses compared to the other three groups before age 26. In addition, participants in the ‘High‐decreasing ADHD, Low emotional problems’ group had lower levels of depression (32.4%) and anxiety diagnoses (10.3%) compared to the ‘Mid‐decreasing ADHD, Increasing emotional problems’ group (46.8% and 20.5%) and the ‘Increasing ADHD, Increasing emotional problems’ group (49.8% and 17.5%). Overall, participants with persistently low symptoms tended to have better mental health outcomes.

**Figure 3:**
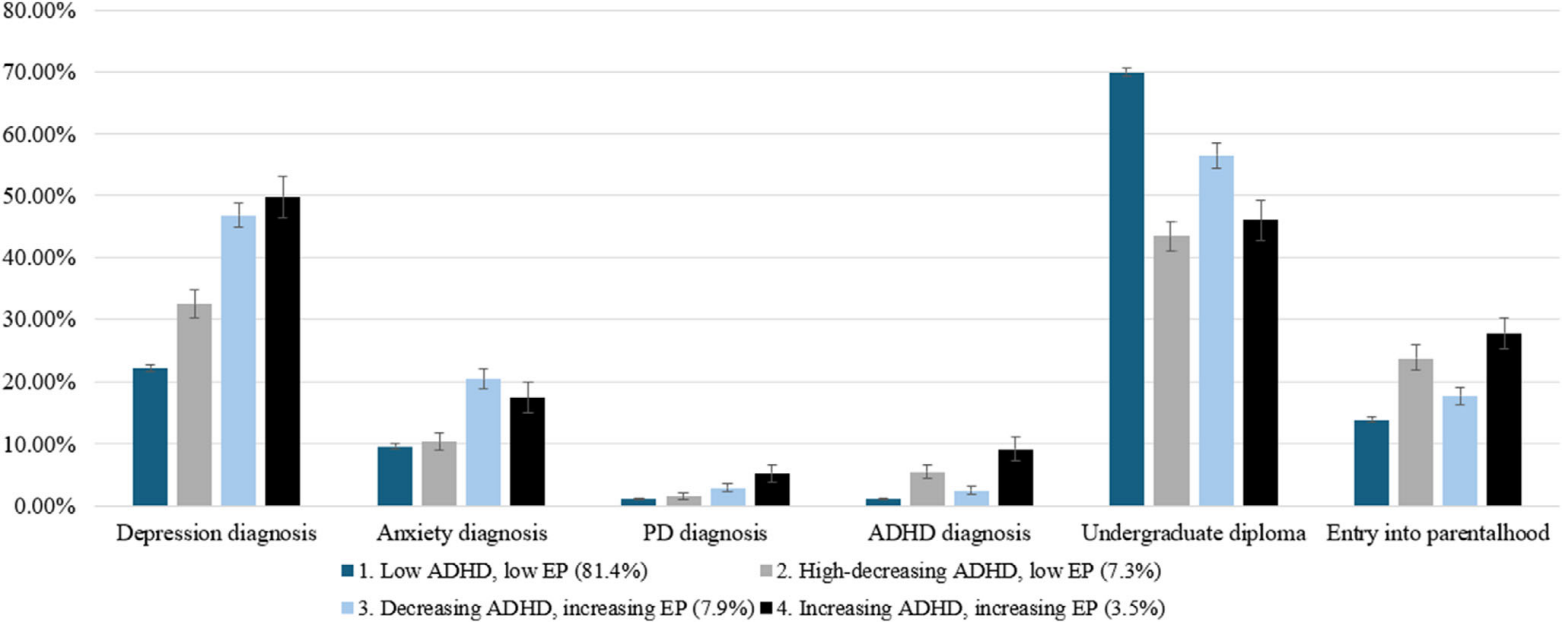
Categorical adult outcomes of the joint trajectory of ADHD and emotional problems based on most likely class membership. All predictors have been standardized to have a mean of zero and *SD* of 1. The error bars represent the standard error of the data. ADHD, attention‐deficit/hyperactivity disorder; EP, emotional problems; PD diagnosis, personality disorder diagnosis

For sociodemographic outcomes, participants in the ‘Low ADHD, Low emotional problems’ group (*M* = 0.09) had the highest adult SES levels compared to the other three groups (*M* = −0.58 to −0.33). The groups with the lowest rate of obtaining an undergraduate diploma were the ‘High‐decreasing ADHD, Low emotional problems’ group and ‘Increasing ADHD, Increasing emotional problems’ group. Participants in the ‘High‐decreasing ADHD, Low emotional problems’ group and the ‘Increasing ADHD, Increasing emotional problems’ group were more likely to have entered parenthood at age 26.

All analyses were rerun with adjustments for attrition using IPW and MI (see Appendices S4–S6). The pattern of results was similar to the unadjusted results presented in the main manuscript (see Tables S9–S11; Figures S5–S7).

### ADHD subdomains

We conducted additional analyses focused on the co‐development of emotional problems and hyperactivity‐impulsivity and emotional problems and inattention. Results are provided in the Supporting Information file (see Tables S12–S17, Figures S8–S13; Appendices S7–S9). In each case, a six‐class model fit the data best. Overall patterns of associations with predictors and outcomes were broadly similar to analyses using the full ADHD scale.

## Discussion

This study identified four joint trajectory classes of ADHD symptoms and emotional problems: ‘Low ADHD, Low emotional problems’, ‘High‐decreasing ADHD, Low emotional problems’, ‘Low ADHD, Increasing emotional problems’, and ‘Increasing ADHD, Increasing emotional problems’. ADHD PGS, externalizing PGS, and early family environment (maternal depression, home chaos, SES) were associated with class membership. The joint trajectories were also associated with psychiatric diagnoses, SES, educational attainment, and entry into parenthood at age 26.

In the joint trajectories, increases in ADHD symptoms were accompanied by rising emotional problems; however, increasing emotional problems were associated with ADHD symptoms that increased, remained stable, or decreased. This pattern aligns with previous research in which ADHD symptoms between ages 5 and 18 predicted later emotional problems, whereas emotional problems did not predict later ADHD (Stern et al., 2020). This could be because ADHD‐related difficulties are associated with further challenges that could contribute to emotional problems.

Consistent with our initial hypotheses, the ‘Low ADHD, Low emotional problems’ group had lower ADHD PGS than the ‘High‐decreasing ADHD, Low emotional problems’ group. The association between ADHD PGS and the high‐decreasing ADHD trajectory may reflect remission patterns, as nearly half of children meet ADHD diagnostic criteria in childhood experience symptom reduction with age (Biederman, Petty, Evans, Small, & Faraone, 2010). In addition, higher ADHD PGS was also observed in the ‘Increasing ADHD, Increasing emotional problems’ group, although this difference was not statistically significant. This pattern may tentatively suggest that the co‐occurrence of ADHD and emotional problems emerging later in development may also show some indication of elevated ADHD genetic liability. Beyond ADHD PGS, the externalizing PGS also distinguished groups, with the ‘Mid‐decreasing ADHD, Increasing emotional problems’ group exhibiting a lower externalizing PGS than both the ‘High‐decreasing ADHD, Low emotional problems’ group and the ‘Increasing ADHD, Increasing emotional problems’ group. This suggests that ADHD symptoms, whether emerging in early childhood or later in development, remain closely linked to externalizing PGS (Brislin et al., 2024; Martínez‐Levy et al., 2024). Notably, the group with joint increases in ADHD and emotional problems showed the numerically highest externalizing PGS values among all groups. While PGS cannot directly reveal the underlying mechanisms, it is possible that the co‐increase of ADHD and emotional problems during development is related to cognitive and emotion regulation processes influenced by genetic risk. Overall, these results suggest that the co‐development of ADHD and emotional problems may be associated with genetic liability for ADHD and externalizing problems. Accordingly, clinical interventions for emotional problems in the neurodivergent groups should consider their distinctive cognitive and emotion regulation processes and the associations between these processes and emotional difficulties. Therefore, overall, both early high levels of ADHD and increases in ADHD with age are associated with genetic risk for externalizing behavior (such as ADHD PGS and externalizing PGS).

Most other psychiatric PGSs (depression, anxiety, internalizing, MDD) did not predict joint trajectories. This may be due to the limited phenotypical variance explained by GWAS‐derived PGSs (Plomin & Von Stumm, 2022). Moreover, the genetic architecture may vary across individuals with different symptom persistence profiles, with a study identifying structural differences among childhood ADHD, persistent ADHD, and late‐diagnosed ADHD (Rajagopal et al., 2022). Psychiatric PGSs may therefore not fully capture long‐term developmental processes.

Maternal depression, family chaos, and early SES were associated with joint trajectories, primarily distinguishing higher‐symptom groups from the ‘Low ADHD, Low emotional problems’ group. These findings align with research suggesting the association between maternal depression and children's internalizing, externalizing, social–emotional, cognitive, language, adaptive outcomes (Rogers et al., 2020; Roubinov et al., 2022), as well as longitudinal changes in ADHD and emotional symptoms (Ahun et al., 2018; Flouri, Ruddy, & Midouhas, 2017). In addition, family chaos and SES were associated with the groups with more severe ADHD and emotional symptoms in the current study. These results are in line with previous research which has shown an association between early‐life adverse environments and internalizing and externalizing behaviors (Dumas, 2015; Lawson, Hook, & Farah, 2018; Marsh, Dobson, & Maddison, 2020). It is also in line with previous TEDS research suggesting children's perceived environment may relate to general psychopathology and that associations may be influenced by gene–environment correlation (Nejand, Malanchini, Voronin, Eley, & Rimfeld, 2026). Specifically, parents' genetic liability may contribute to home chaos, while children's genetic liability may evoke disorganized parenting. This bidirectional influence warrants further study.

In terms of mental health outcomes, as expected, the ‘Low ADHD, Low emotional problems’ group had the lowest lifetime rates of depression, anxiety, or ADHD by age 26, followed by the ‘High‐decreasing ADHD, Low emotional problems’ group. The latter showed significantly or non‐significantly lower rates of depression, anxiety, and personality disorders than the ‘Increasing ADHD, Increasing emotional problems’ and ‘Mid‐decreasing ADHD, Increasing emotional problems’ groups, suggesting that symptom‐increasing trajectories may persist into later stages.

In addition, both the ‘High‐decreasing ADHD, Low emotional problems’ and ‘Increasing ADHD, Increasing emotional problems’ groups showed lower educational attainment, higher rates of early parenthood, and numerically lower adult SES. These findings suggest that early high ADHD symptoms affect adult outcomes even if symptoms later decrease. Access to higher education depends largely on childhood and adolescent academic performance. ADHD symptoms such as rule‐breaking and inattention are associated with school dropout (Breslau, 2010; Fredriksen et al., 2014), influencing higher education and later SES (Slominski, Sameroff, Rosenblum, & Kasser, 2011). Increasing ADHD and emotional symptoms were also associated with poorer academic outcomes, consistent with previous findings (Patalay, Deighton, Fonagy, & Wolpert, 2015; Pingault et al., 2011). This may stem from the accumulating challenges posed by persistently escalating symptoms. Therefore, overall, attention to groups who may face adverse socioeconomic or educational outcomes associated with ADHD and emotional problems should not be limited to individuals with high current symptom levels. Instead, they should also include those who had high symptom levels earlier in development, as they likewise face challenges in educational, occupational, and mental health.

It is worth noting that 26 years old reflects relatively early adulthood. Many important consequences (e.g. long‐term occupational trajectories and health burden) may only manifest later in life when they have been living with mental health symptoms and neurodevelopmental conditions for a long time (de Sousa et al., 2025; Foulds et al., 2025; Kessler et al., 2005). Therefore, future research should explore longer‐term outcomes of ADHD and emotional problems.

## Limitations

This is the first study to examine the joint developmental trajectories of ADHD symptoms and emotional problems from childhood to adulthood by using a large, representative sample that included longitudinal data from more than 25,000 individuals in England and Wales, spanning childhood to early adulthood. A range of predictors and outcomes were also explored in this study, including genetic factors, mental health, environmental influences, and sociodemographic variables, providing an understanding of the genetic and environmental correlates of the co‐development of ADHD symptoms and emotional problems.

There are several limitations of this study. First, while several results are statistically significant, the effect sizes are often modest. Small effects may have limited practical impact when applied to interventions targeting individual cases. However, when considered at a broader level, such as school‐wide screening, even relatively small effect sizes can translate into meaningful implications because of the large number of individuals affected. Second, it is worth noting that the TEDS cohort is predominantly White (91.9%), UK‐based, and limited to individuals born in the 1990s. This may limit the generalizability of findings to more diverse cultural and clinical populations. Third, parent reports can introduce biases; for example, in twin studies, parent reports of ADHD in dizygotic twins often show contrast effects, meaning a tendency to emphasize symptom differences between the two children (Rietveld, Posthuma, Dolan, & Boomsma, 2003). However, analyses in the TEDS sample have shown that polygenic prediction of ADHD symptoms does not differ significantly across parent and self‐reports. This suggests that ADHD symptoms reported by parents do capture behavior influenced by the child's genome (independent of parent genome), and this is not simply a reflection of reporter bias (Bright et al., 2025). Lastly, common method variance may be another concern due to the similarity of reporters. In the current study, because symptoms of ADHD and emotional problems were both reported by parents, this may have inflated the association between them.

## Implications

The findings of this study have important implications. Our results suggest that it is essential to recognize and address emotional difficulties in children with ADHD symptoms, particularly those with increasing ADHD symptoms over time, as these increases are often accompanied by increased emotional problems. In addition, since maternal depression and family chaos are associated with symptom trajectories, family‐based interventions may be beneficial. For example, promoting a supportive home environment by addressing parental mental health and reducing family chaos may help mitigate children's symptoms. Conversely, families of children with high levels of psychopathological symptoms may also benefit from interventions that help them manage potential stressors, which in turn can reduce family chaos. Furthermore, we found that high ADHD symptoms early in life, as well as increasing ADHD and emotional problems, were associated with a lower likelihood of obtaining higher‐education qualifications. This suggests that education interventions should not be limited to those who are currently affected by severe ADHD or emotional problems, but also to individuals who showed high levels of ADHD symptoms early in development, as they may continue to face substantial academic challenges even if their ADHD symptoms diminish with age.

## Conclusion

ADHD and emotional problems exhibit both co‐occurring and independent developmental patterns. In groups with increasing ADHD symptoms, emotional problems also tended to show an upward trend. In contrast, among groups with rising emotional problems, ADHD symptoms may increase, remain stable, or decrease. Higher co‐occurring symptom levels were associated with ADHD PGSs, externalizing PGS, and childhood environmental predictors such as maternal depression, family chaos, and low SES. Groups with high symptom levels generally had higher rates of mental health diagnoses in adulthood. In particular, individuals in symptom‐increasing groups faced elevated risks of mental health diagnoses and lower SES compared to other groups. High ADHD symptom‐decreasing and symptom‐increasing groups were more likely to report low educational attainment, emphasizing the impact of childhood ADHD on academic outcomes and the importance of targeted interventions. This study makes an important contribution to understanding the co‐developmental patterns of ADHD and emotional problems, as well as their associated predictors and developmental outcomes. Future research could build on these findings to further investigate the generalizability of the results across different cultural contexts.

## Ethical considerations

Ethical approval for TEDS was provided by the King's College London Ethics Committee (reference: PNM/09/10‐104). Written informed consent was obtained from parents prior to data collection and from twins themselves from age 16 onward. The use of anonymized data for academic purposes did not require additional ethical approval.

**Key points**

**What's known?**

- ADHD symptoms and emotional problems frequently co‐occur.

**What's new?**

- This study identified four distinct trajectories of ADHD symptoms and emotional problems from ages 4 to 21. Compared with participants in the consistently low‐symptom class, those with initially high or persistently high ADHD symptoms or emotional problems had higher ADHD and externalizing polygenic scores, lower SES, greater household chaos, and higher maternal depressive symptoms at baseline. At age 26, participants in the consistently low‐symptom class had higher educational attainment and fewer lifetime diagnoses of depression and anxiety than those in the other classes.

**What's relevant?**

- These findings can help clinicians and educators identify children at risk for persistent ADHD and emotional problems, enabling timely support to improve long‐term outcomes.

## Supporting information


**Table S1** Baseline characteristics of study cohort (*n* = 27,890).
**Appendix S1**. Notes on the correlations between ADHD subdomains and emotional problems.
**Table S2**. The correlation of ADHD subdomains and emotional problems measured at different time points.
**Appendix S2**. Notes on the single‐phenotype latent growth models of ADHD, inattention, hyperactivity‐impulsivity, and emotional problems.
**Table S3**. Relative fit indices for 1 through 5 class models for the trajectories of ADHD.
**Table S4**. Relative fit indices for 1 through 5 class models for the trajectories of hyperactivity‐impulsivity.
**Table S5**. Relative fit indices for 1 through 5 class models for the trajectories of inattention.
**Table S6**. Relative fit indices for 1 through 5 class models for the trajectories of emotional problems.
**Appendix S3**. Validation measures for the joint trajectory classes.
**Table S7**. Descriptive statistics of several validation variables for the joint trajectories of ADHD and emotional problems.
**Table S8**. Pairwise comparisons of several validation variables for the joint trajectories of ADHD and emotional problems.
**Appendix S4**. Attrition.
**Appendix S5**. Inverse probability weighting and multiple imputation.
**Appendix S6**. The joint trajectory of ADHD and emotional problems when using a subsample with inverse probability weighting and multiple imputations.
**Table S9**. Relative fit indices for 1 through 4 class models for joint trajectories of ADHD and emotional problems by using the complete sample with inverse probability weighting.
**Table S10**. Descriptive statistics of predictor and outcome variables for the joint trajectory of ADHD and emotional problems based on most likely latent class membership by using a sample with at least two time points of measures.
**Table S11**. Pairwise comparisons of predictor and outcome variables for the joint trajectory of ADHD and emotional problems across classes in the three‐step method by using a sample with at least two time points of measures.
**Appendix S7**. Joint trajectories of hyperactivity‐impulsivity and emotional problems.
**Appendix S8**. Joint trajectories of inattention and emotional problems.
**Appendix S9**. The differences in the joint trajectories of ADHD subdomains and emotional problems.
**Table S12**. Relative fit indices for 1 through 5 class models for the joint trajectories of hyperactivity‐impulsivity and emotional problems.
**Table S13**. Relative fit indices for 1 through 5 class models for the joint trajectories of inattention and emotional problems.
**Table S14**. Descriptive statistics of predictor and outcome variables for the joint trajectory of hyperactivity‐impulsivity and emotional problems based on most likely latent class membership.
**Table S15**. Pairwise comparisons of predictor and outcome variables for the joint trajectory of hyperactivity‐impulsivity and emotional problems across classes in the three‐step method.
**Table S16**. Descriptive statistics of predictor and outcome variables for the joint trajectory of inattention and emotional problems based on most likely latent class membership.
**Table S17**. Pairwise comparisons of predictor and outcome variables for the joint trajectory of inattention and emotional problems across classes in the three‐step method.
**Figure S1**. Trajectories of ADHD.
**Figure S2**. Trajectories of hyperactivity‐impulsivity.
**Figure S3**. Trajectories of inattention.
**Figure S4**. Trajectories of emotional problems.
**Figure S5**. Joint trajectories of ADHD and emotional problems by using subsample with inverse probability weighting.
**Figure S6**. Predictors for the joint trajectory of ADHD and emotional problems based on the most likely latent class membership by using subsample with inverse probability weighting.
**Figure S7**. Categorical outcomes of the joint trajectory of ADHD and emotional problems based on most likely latent class membership by using subsample with inverse probability weighting.
**Figure S8**. Joint trajectories of hyperactivity‐impulsivity and emotional problems
**Figure S9**. Predictors for the joint trajectory of hyperactivity‐impulsivity and emotional problems based on most likely latent class membership.
**Figure S10**. Categorical adult outcomes of the joint trajectory of ADHD and emotional problems based on most likely class membership
**Figure S11**. Joint trajectories of inattention and emotional problems.

## Data Availability

Data for this study came from the Twins Early Development Study (TEDS). Researchers can apply for managed access to TEDS at: https://www.teds.ac.uk/researchers/teds‐data‐access‐policy.
