# Supplementary material for: Co‐development of ADHD symptoms and emotional problems from childhood to adulthood: predictors and developmental outcomes
Source: J Child Psychol Psychiatry. 2026 Jun 30;67(12):1994–2007. doi: 10.1111/jcpp.70196 (PMC13745783; doi:10.1111/jcpp.70196)
Supplement: Supplementary file 1 [file JCPP-67-1994-s001.docx]

**Co-development of ADHD symptoms and emotional problems from childhood to adulthood: predictors and developmental outcomes**

**Supporting Information**

| **Table S1**. Baseline Characteristics of Study Cohort (n = 27890) | | |
| --- | --- | --- |
| **Demographic variables** | N | % |
| Sex, female | 13971 | 50.1% |
| Race, white | 25126 | 91.9% |
| Race, other | 2212 | 8.1% |
|  | Mean | SD |
| Age 4 | 4.02 | 0.13 |
| Age 7 | 7.12 | 0.27 |
| Age 8 | 7.93 | 0.52 |
| Age 9 | 9.02 | 0.28 |
| Age 12 | 11.31 | 0.72 |
| Age 14 | 14.07 | 0.57 |
| Age 16 | 16.32 | 0.68 |
| Age 21 | 22.3 | 0.93 |
| Age 26 | 26.42 | 0.92 |
| **Main variables** | N | % |
| Depression diagnosis | 2017 | 25.9% |
| Anxiety diagnosis | 841 | 10.8% |
| PD diagnosis | 105 | 1.3% |
| ADHD diagnosis | 135 | 1.7% |
| Undergraduate diploma | 5222 | 66.6% |
| Entry into parenthood at 26 | 1279 | 15.3% |
|  | Mean | SD |
| ADHD PGS | 1.08 | 0.56 |
| Anxiety PGS | -1.38 | 0.38 |
| Depression PGS | 0.52 | 0.31 |
| EA PGS | -0.46 | 0.29 |
| Externalizing PGS | 0.18 | 0.18 |
| MDD PGS | -5.20 | 0.12 |
| Maternal depression | 7.58 | 4.70 |
| Family chaos | -0.02 | 0.99 |
| SES | 0.08 | 1.00 |
| Conners inattention | 6.60 | 6.16 |
| SDQ_EP | 2.88 | 2.67 |
| SDQ_hyper | 2.93 | 2.18 |
| SES | 0.03 | 0.98 |

Note: The percentage refers to the proportion among participants with available data.

EP, emotional problems; ADHD, attention-deficit/Hyperactivity disorder; PGS, polygenic risk scores; EA, educational attainment; SES, socioeconomic Status; MDD, major depressive disorder; PD, personality disorder;

SDQ_EP: SDQ emotional problems scale; SDQ_hyper: SDQ hyperactivity scale.

**Appendix S1**. Notes on the correlations between ADHD subdomains and emotional problems

As is shown in Table S2, measures of ADHD subdomains and emotional problems correlated with each other within and across timepoints (*r* = .10 to .72). On average, hyperactivity-impulsivity symptoms showed a declining trend with age from childhood (M = 5.79) to adulthood (M = 2.53). Inattention symptoms initially show an increasing trend from age 8 (M = 5.39) to 12 (M = 5.57), followed by a continuous decline into adulthood (M = 3.96).

| **Table S2**. The correlation of ADHD subdomains and emotional problems measured at different time points | | | | | | | | | | | | |
| --- | --- | --- | --- | --- | --- | --- | --- | --- | --- | --- | --- | --- |
| Symptoms | M(SD) | 1 | 2 | 3 | 4 | 5 | 6 | 7 | 8 | 9 | 10 |  |
| Hyperactivity-impulsivity | |  |  |  |  |  |  |  |  |  |  |  |
| 1 age4 EP | 1.39(1.44) | 1 |  |  |  |  |  |  |  |  |  |  |
| 2 age7 EP | 2.20(1.84) | .38*** | 1 |  |  |  |  |  |  |  |  |  |
| 3 age9 EP | 1.72(1.87) | .36*** | .51*** | 1 |  |  |  |  |  |  |  |  |
| 4 age12 EP | 1.82(1.93) | .31*** | .45*** | .52*** | 1 |  |  |  |  |  |  |  |
| 5 age21 EP | 2.37(2.29) | .19*** | .30*** | .37*** | .39*** | 1 |  |  |  |  |  |  |
| 6 age8 Hyper | 5.79(5.09) | .15*** | .18*** | .21*** | .20*** | .12*** | 1 |  |  |  |  |  |
| 7 age12 Hyper | 4.29(4.39) | .15*** | .15*** | .22*** | .26*** | .13*** | .68*** | 1 |  |  |  |  |
| 8 age14 Hyper | 3.55(4.15) | .12*** | .14*** | .17*** | .19*** | .13*** | .57*** | .69*** | 1 |  |  |  |
| 9 age16 Hyper | 2.60(3.49) | .13*** | .14*** | .19*** | .20*** | .17*** | .47*** | .58*** | .69*** | 1 |  |  |
| 10 age21 Hyper | 2.53(3.74) | .10*** | .11*** | .14*** | .15*** | .23*** | .39*** | .47*** | .53*** | .55*** | 1 |  |
| Inattention | |  |  |  |  |  |  |  |  |  |  |  |
| 1 age4 EP | 1.39(1.44) | 1 |  |  |  |  |  |  |  |  |  |  |
| 2 age7 EP | 2.20(1.84) | .38*** | 1 |  |  |  |  |  |  |  |  |  |
| 3 age9 EP | 1.72(1.87) | .36*** | .51*** | 1 |  |  |  |  |  |  |  |  |
| 4 age12 EP | 1.82(1.93) | .31*** | .45*** | .52*** | 1 |  |  |  |  |  |  |  |
| 5 age21 EP | 2.37(2.29) | .19*** | .30*** | .37*** | .39*** | 1 |  |  |  |  |  |  |
| 6 age8 Inattention | 5.39(5.16) | .15*** | .20*** | .24*** | .22*** | .15*** | 1 |  |  |  |  |  |
| 7 age12 Inattention | 5.57(5.14) | .13*** | .15*** | .23*** | .29*** | .15*** | .66*** | 1 |  |  |  |  |
| 8 age14 Inattention | 5.11(5.18) | .13*** | .13*** | .18*** | .21*** | .15*** | .57*** | .71*** | 1 |  |  |  |
| 9 age16 Inattention | 4.21(4.89) | .11*** | .14*** | .19*** | .21*** | .19*** | .49*** | .61*** | .72*** | 1 |  |  |
| 10 age21 Inattention | 3.96(4.53) | .10*** | .13*** | .16*** | .18*** | .31*** | .39*** | .46*** | .53*** | .56*** | 1 |  |

Note: ^*^*p*<0.05, ^**^*p*<0.01, ^***^*p*<0.001.

EP, emotional problems; Hyper, hyperactivity-impulsivity.

**Appendix S2**. Notes on the single-phenotype latent growth models of ADHD, inattention, hyperactivity-impulsivity, and emotional problems

We used single-phenotype latent growth models to capture the developmental trajectories of ADHD symptoms, inattention, hyperactivity-impulsivity, and emotional problems from early childhood to adulthood. We began by constructing both linear and quadratic latent growth models to examine how these traits change over time. For emotional problems and hyperactivity, quadratic models resulted in a variance covariance matrix that was not positive definite. Therefore, we only consider linear models in the following analyses.

For the trajectory models of ADHD, the 4-class model provided the best fit (see Table S3 and Figure S1). The following categories were identified: low ADHD, high ADHD, decreasing ADHD, and increasing ADHD. Similarly, for the hyperactivity-impulsivity subdomian, the 4-class model provided the best fit (see Table S4 and Figure S2). It comprised low hyperactivity-impulsivity, high hyperactivity-impulsivity, decreasing hyperactivity-impulsivity, and increasing hyperactivity-impulsivity. However, for the inattention subdomain, the 8-class model was the best-fitting (see Table S5 and Figure S3). This model included a low inattention group (class 1), mid inattention group (class 5), high inattention group (class 7), decreasing inattention groups (class 4; class 3) , and increasing inattention groups (class 8; class 4; class 2).

The 4-classes trajectories of emotional problems provided the best fit to the data (see Table S6 and Figure S4), comprising a low emotional problems group, a high emotional problems group, a decreasing emotional problems group, and an increasing emotional problems group were detected.

| **Table S3**. Relative fit indices for 1 through 5 class models for the trajectories of ADHD | | | | | |
| --- | --- | --- | --- | --- | --- |
| Number of classes | AIC | BIC | ABIC | LMRT | Entropy |
| 1 | 341754 | 341816 | 341791 | - | - |
| 2 | 336807 | 336892 | 336857 | 0 | 0.882 |
| 3 | 333957 | 334065 | 334020 | 0 | 0.865 |
| **4** | **332500** | **332631** | **332577** | **0.005** | **0.837** |
| 5 | 331561 | 331716 | 331652 | 0.287 | 0.823 |

Note: AIC: Akaike information criterion (AIC); BIC: Bayesian information criterion; ABIC: Adjusted Bayesian information criterion; LMRT: Lo–Mendel–Rubin test.

| **Table S4**. Relative fit indices for 1 through 5 class models for the trajectories of hyperactivity-impulsivity | | | | | |
| --- | --- | --- | --- | --- | --- |
| Number of classes | AIC | BIC | ABIC | LMRT | Entropy |
| 1 | 275874 | 275936 | 275910 | - | - |
| 2 | 270294 | 270379 | 270344 | 0 | 0.900 |
| 3 | 266972 | 267080 | 267035 | 0 | 0.877 |
| **4** | **265160** | **265292** | **265238** | **0.069** | **0.857** |
| 5 | 264033 | 264188 | 264124 | 0.536 | 0.830 |

Note: AIC: Akaike information criterion; BIC: Bayesian information criterion; ABIC: Adjusted Bayesian information criterion; LMRT: Lo–Mendel–Rubin test.

| **Table S5**. Relative fit indices for 1 through 5 class models for the trajectories of inattention | | | | | |
| --- | --- | --- | --- | --- | --- |
| Number of classes | AIC | BIC | ABIC | LMRT | Entropy |
| 1 | 292310 | 292371 | 292346 | - | - |
| 2 | 288278 | 288263 | 288229 | 0 | 0.887 |
| 3 | 285013 | 285121 | 285077 | 0 | 0.854 |
| 4 | 283848 | 283979 | 283925 | 0.002 | 0.830 |
| 5 | 282949 | 283103 | 283040 | 0.010 | 0.805 |
| 6 | 282235 | 282413 | 282340 | 0 | 0.791 |
| 7 | 281784 | 281985 | 281903 | 0.032 | 0.778 |
| **8** | **281235** | **281459** | **281367** | **0.01** | **0.759** |
| 9 | 280929 | 281176 | 281074 | 0.06 | 0.760 |

Note: AIC: Akaike information criterion; BIC: Bayesian information criterion; ABIC: Adjusted Bayesian information criterion; LMRT: Lo–Mendel–Rubin test.

| **Table S6**. Relative fit indices for 1 through 5 class models for the trajectories of emotional problems | | | | | |
| --- | --- | --- | --- | --- | --- |
| Number of classes | AIC | BIC | ABIC | LMRT | Entropy |
| 1 | 229673 | 229736 | 229711 | - | - |
| 2 | 226767 | 226853 | 226819 | 0 | 0.773 |
| 3 | 224946 | 225056 | 225011 | 0 | 0.716 |
| **4*** | **224003** | **224137** | **224083** | **0** | **0.706** |
| 5 | 223522 | 223680 | 223616 | 0.066 | 0.695 |

Note: AIC: Akaike information criterion (AIC); BIC: Bayesian information criterion; ABIC: Adjusted Bayesian information criterion; LMRT: Lo–Mendel–Rubin test.

*These trajectories significantly overlap with previously identified emotional symptom trajectories reported in Palaiologou et al. (2025). The main difference is that the four-class model identified in our paper reports a high emotional problems class, whereas Palaiologou et al 2025 report a steep adolescent-increasing class. However, a persistent class emerged in their five-class solution, suggesting that the discrepancy reflects model specification rather than a substantive inconsistency. The two studies followed different methodologies and thus variations in the modelling approaches, sample sizes, raters and age ranges likely explain the observed variation in the class solutions.

**Appendix S3. Validation measures for the joint trajectory classes**

The Conners questionnaire, hyperactivity measured by the SDQ, and emotional problems measured by the SDQ at age 26 were used as validation measures for the joint trajectory classes (see Table S7-S8). The results showed that participants in the “Low ADHD, Low emotional problems” group had the lowest scores in Conners inattention, SDQ emotional problems and SDQ Hyperactivity compared to the other three groups at age 26. Participants in the “High-decreasing ADHD, Low emotional problems” group (M = 0.08) also had lower SDQ emotional problems scores than those in the “Mid-decreasing ADHD, Increasing emotional problems” group (M = 0.55) and the “Increasing ADHD, Increasing emotional problems” group (M = 0.37). In addition, participants in the “Increasing ADHD, Increasing emotional problems” reported the highest level of inattention symptoms in Conners as well as highest hyperactivity in SDQ. The results supported the validity of the trajectory classification.

| **Table S7**. Descriptive statistics of several validation variables for the joint trajectories of ADHD and emotional problems | | | | | |  |
| --- | --- | --- | --- | --- | --- | --- |
| Trajectory group (M, SD/N, %) | | | | | |  |
|  | n | 1. Low ADHD, low EP (81.4%) | 2. High-decreasing ADHD, low EP （7.3%） | 3. Mid-decreasing ADHD, increasing EP（7.9%） | 4. Increasing ADHD, increasing EP (3.5%） |  |
| Conners inattention | 7303 | -0.08(0.95) | 0.28(1.12) | 0.25(1.10) | 0.77(1.29) |  |
| SDQ_EP | 7385 | -0.08(0.96) | 0.08(1.05) | 0.55(1.07) | 0.37(1.10) |  |
| SDQ_hyper | 7385 | -0.06(0.97) | 0.26(1.01) | 0.23(1.07) | 0.56(1.11) |  |

Note: All predictors and outcomes have been standardized to have a mean of zero and SD of 1.EP, emotional problems; ADHD, attention-deficit/hyperactivity disorder;

SDQ_EP: SDQ emotional problems scale; SDQ_hyper: SDQ hyperactivity scale.

M, mean; SD, standard deviation;

Trajectory group (M, SD): The mean and standard deviation of predictors in each trajectory group based on the most likely latent class membership.

Trajectory group (N, %): The number and proportion of participants in each trajectory group based on the most likely latent class membership.

*Descriptive statistics based on “most likely classes” may be inconsistent with the results of subsequent pairwise comparisons using the three-step method, as the latter accounts for uncertainty in class membership.

Note: 1. Low ADHD, low EP (81.4%); 2. High-decreasing ADHD, low EP (7.3%); 3. Mid-decreasing ADHD, increasing EP (7.9%); 4. Increasing ADHD, increasing EP (3.5%).

Baseline SES was controlled for when examining the associations between trajectory classes and validation variables.

EP, emotional problems; ADHD, attention-deficit/hyperactivity disorder;

SDQ_EP: SDQ emotional problems scale; SDQ_hyper: SDQ hyperactivity scale.

| **Table S8**. Pairwise comparisons of several validation variables for the joint trajectories of ADHD and emotional problems | | | | | | |
| --- | --- | --- | --- | --- | --- | --- |
| Group comparation (Chi-square) | | | | | | |
|  | 2 VS. 1 | 3 VS. 1 | 4 VS. 1 | 3 VS. 2 | 4 VS. 2 | 4 VS. 3 |
| **Conners inattention** | 30.20* | 48.41* | 90.50* | 0.003 | 20.54* | 24.79* |
| **SDQ_EP** | 6.77 | 181.81* | 38.03* | 50.75* | 10.79* | 5.99 |
| **SDQ_hyper** | 30.52* | 40.92* | 67.60* | 0 | 10.62* | 12.46* |

**Appendix S4. Attrition**

At the first measurement occasions of the joint trajectories (age 4 years for emotional problems and age 8 years for ADHD symptoms), 15182 (58.7%) of TEDS participants provided data on emotional problems and 12884 (49.8%) participants provided data on ADHD symptoms. 12625 (48.8%) participants had provided at least two measurements for both ADHD and emotional problems. 3090 (11.9%) participants had complete data on emotional problems across all waves, and 3744 (14.5%) participants had complete data on ADHD symptoms across all waves. A total of 2004 (7.7%) participants had complete data on both emotional problems and ADHD symptoms across all waves.

**Appendix S5. Inverse probability weighting and multiple imputation**

To examine whether non-random missingness could bias the results obtained using FIML in the main analyses, a sensitivity analysis based on participants with trajectory data was conducted. For missingness in the trajectory variables, multiple imputation (MI) could lead to inconsistencies in trajectory classification across imputed datasets; therefore, we used an inverse probability weighting (IPW) approach to account for non-random missingness. Specifically, IPWs were constructed based on the probability that participants had at least two time points of both ADHD and emotional problems (N = 12625). This approach was used for two main reasons. First, to account for the non-random inclusion of participants in the trajectory analysis within the TEDS sample. Second, to address the potential bias arising from including individuals with only a single measurement when fitting trajectory models using FIML, as such individuals contribute limited information to the estimation of developmental patterns. Specifically, the predictors used to construct the IPW included a series of variables with relatively low levels of missingness that were independently associated with whether participants had at least two time points of measurement for both ADHD and emotional problems. The categorical variables included: ethnicity, *χ*²(1) = 58.03, *p* < .001; household type, *χ*²(3) = 126.36, *p* < .001; female parent’s highest qualification, *χ*²(7) = 642.28, *p* < .001; male parent’s highest qualification, *χ*²(7) = 331.68, *p* < .001; male parent’s social class/employment level, *χ*²(5) = 212.38, *p* < .001. The continuous variables included: age of the natural mother at the twins’ birth (*p* < .001); age of the natural father at birth (*p* < .001); childhood SES (*p* < .001); ADHD PGS (*p* < .001); depression PGS (*p* = .003); educational attainment PGS (*p* < .001); externalizing PGS (*p* < .001); and MDD PGS (*p* = .03). Accordingly, these variables were included as predictors in the construction of the IPWs. A small amount of missing data in the continuous indicators used to construct the weights was addressed using single mean or mode imputation. Model fit for the missingness model was satisfactory, with no indication of poor fit according to the Hosmer–Lemeshow test (*χ*²(8) = 14.62, *p* = 0.07). In the included sample, the final inverse probability weights ranged from 0.64 to 2.24.

Subsequently, MI was applied to the trajectory predictors (psychiatric polygenic scores and early environmental factors) and outcomes (mental health outcomes and socioeconomic status outcomes) among participants with at least two time points of measures of both ADHD and emotional problems, generating 250 imputed datasets. The following variables were included in the MI models: all predictors and outcomes; sociodemographic variables (e.g., sex and ethnicity); emotional problems and ADHD symptoms. Overall, the pattern of results after applying subsample analysis with IPW to the trajectory and MI to the predictors and outcomes was highly similar to those from the primary analyses and did not alter the interpretation of findings.

**Appendix S6. The joint trajectory of ADHD and emotional problems when using subsample with inverse probability weighting and multiple imputations**

For the joint ADHD and emotional problems model, although the LMRT suggested that a five-class solution could be considered, the additional class identified in the five-class model, “Mid-decreasing ADHD, Low emotional problems,” largely overlapped with the existing classes. Therefore, for validation of the original findings, we still adopted the four-class solution as the best-fitting model. The four-class solution included the following categories: "Low ADHD, Low emotional problems", "High-decreasing ADHD, Low emotional problems", "Mid-decreasing ADHD, Increasing emotional problems" and "Increasing ADHD, Increasing emotional problems". Overall, the trajectory patterns were similar to those observed in the four-class solution reported in the original study (see Table S9; Figure S5).

Regarding predictors (see Table S10, Figure S6), similarly, ADHD PGS and Externalizing PGS were elevated in either or both the “High-decreasing ADHD, Low emotional problems” group and the “Increasing ADHD, Increasing emotional problems” group. Additionally, early adverse environments, including higher levels of maternal depression, family chaos, and low SES, were significantly more prevalent in the “High-decreasing ADHD, Low emotional problems” group compared with the “Low ADHD, Low emotional problems” group, which is consistent with the findings reported in the original analysis.

With respect to outcomes (see Table S11, Figure S7), the results based on the subsample with IPW were similar to those observed in our main analysis as well. Specifically, participants in the “Low ADHD, Low emotional problems” group had lower rates of psychiatric diagnoses and were more likely to obtain an undergraduate degree than those in the other three groups. Participants in both the “High-decreasing ADHD, Low emotional problems” and “Increasing ADHD, Increasing emotional problems” groups had lower rates of obtaining an undergraduate degree and higher rates of entry into parenthood by age 26 than those in the other two groups.

| **Table S9**. Relative fit indices for 1 through 4 class models for joint trajectories of ADHD and emotional problems by using complete sample with inverse probability weighting | | | | | |
| --- | --- | --- | --- | --- | --- |
| Number of classes | AIC | BIC | ABIC | LMRT | Entropy |
| 1 | 488787 | 488913 | 488859 | - | - |
| 2 | 484913 | 485076 | 485007 | 0.000 | 0.891 |
| 3 | 482081 | 482282 | 482196 | 0.000 | 0.885 |
| **4** | **480753** | **480992** | **480890** | **0.003** | **0.826** |
| 5 | 479664 | 479939 | 479822 | 0.040 | 0.818 |
| 6 | 478949 | 479262 | 479128 | 0.664 | 0.823 |

Note: AIC: Akaike information criterion (AIC); BIC: Bayesian information criterion; ABIC: Adjusted Bayesian information criterion; LMRT: Lo–Mendel–Rubin test.

| **Table S10.** Descriptive statistics of predictor and outcome variables for the joint trajectory of ADHD and emotional problems based on most likely latent class membership by using a sample with at least two time points of measures | | | | | |  |
| --- | --- | --- | --- | --- | --- | --- |
| Trajectory group (M, SD/%) | | | | | |  |
|  |  | 1. Low ADHD, low EP (77.4%) | 2. High-decreasing ADHD, low EP (8.9%) | 3. Mid-decreasing ADHD, increasing EP (9.6%) | 4. Increasing ADHD, increasing EP (4.2%) |  |
|  |  |  |  |  |  |  |
| Predictors(M, SD) | ADHD PGS | -0.09(1.00) | 0.21(0.99) | 0.13(1.02) | 0.20(1.00) |  |
|  | Anxiety PGS | 0.01(1.00) | 0.06(1.05) | -0.05(0.97) | -0.05(1.00) |  |
|  | Depression PGS | -0.07(0.99) | 0.01(1.01) | 0.10(1.01) | 0.36(1.03) |  |
|  | EA PGS | 0.13(0.99) | -0.21(1.00) | -0.05(1.04) | -0.01(0.96) |  |
|  | Externalizing PGS | -0.09(0.99) | 0.14(1.00) | -0.1(1.01) | 0.28(0.99) |  |
|  | MDD PGS | -0.07(0.99) | -0.08(0.97) | 0.04(1.06) | 0.01(1.03) |  |
|  | Maternal depression | -0.14(0.98) | 0.32(1.06) | 0.30(1.04) | 0.38(1.03) |  |
|  | Family chaos | -0.16(0.97) | 0.39(0.97) | 0.21(0.92) | 0.31(1.00) |  |
|  | SES | 0.09(0.99) | -0.34(0.97) | -0.13(1.05) | -0.21(0.99) |  |
| Continuous outcomes(M, SD) | SES | 0.13(0.92) | -0.27(1.17) | -0.57(1.28) | -0.41(1.12) |  |
|  | Depression diagnosis | 21.40% | 23.30% | 51.50% | 47.20% |  |
|  | Anxiety diagnosis | 8.70% | 6.50% | 23.60% | 14.70% |  |
|  | PD diagnosis | 1.00% | 0.70% | 2.70% | 3.20% |  |
| Categorical outcomes(N, %) | ADHD diagnosis | 1.40% | 5.60% | 2.80% | 11.60% |  |
|  | Undergraduate diploma | 68.10% | 37.70% | 55.50% | 47.20% |  |
|  | Entry into parenthood | 9.00% | 17.70% | 9.60% | 16.00% |  |

Note: Trajectory group M(SD)/%: The descriptive statistics in this section are based on the means, variances, and proportions of the non-imputed predictors and outcomes within the trajectory classes obtained using inverse probability weighting.

EP, emotional problems; ADHD, attention-deficit/hyperactivity disorder; PGS, polygenic risk scores; EA, educational attainment; SES, socioeconomic Status; MDD, major depressive disorder; PD, personality disorder.

| **Table S11**. Pairwise comparisons of predictor and outcome variables for the joint trajectory of ADHD and emotional problems across classes in the three-step method by using using a sample with at least two time points of measures | | | | | | | |
| --- | --- | --- | --- | --- | --- | --- | --- |
| Group comparation (OR, SE/Chi-square) | | | | | | | |
|  |  | 2 VS. 1 | 3 VS. 1 | 4 VS. 1 | 3 VS. 2 | 4 VS. 2 | 4 VS. 3 |
|  |  |  |  |  |  |  |  |
| Predictors(OR, SE) | **ADHD PGS** | 1.18(0.06)* | 1.13(0.05) | 1.15(0.08) | 0.95(0.06) | 0.98(0.08) | 1.02(0.08) |
|  | Anxiety PGS | 1.00(0.05) | 0.95(0.04) | 1.01(0.07) | 0.95(0.06) | 1.02(0.08) | 1.08(0.08) |
|  | Depression PGS | 1.04(0.06) | 1.10(0.05) | 1.10(0.09) | 1.05(0.07) | 1.06(0.10) | 1.01(0.09) |
|  | EA PGS | 0.95(0.05) | 0.96(0.04) | 0.94(0.06) | 1.01(0.06) | 0.99(0.08) | 0.98(0.07) |
|  | Externalizing PGS | 1.14(0.06) | 0.91(0.04) | 1.21(0.08) | 0.80(0.05)* | 1.06(0.08) | 1.33(0.10)* |
|  | MDD PGS | 0.98(0.05) | 1.04(0.05) | 1.01(0.08) | 1.07(0.07) | 1.04(0.09) | 0.97(0.08) |
|  | **Maternal depression** | 1.21(0.06)* | 1.20(0.06)* | 1.17(0.08) | 0.99(0.07) | 0.96(0.07) | 0.97(0.08) |
|  | **Family chaos** | 1.30(0.07)* | 1.12(0.05) | 1.27(0.08)* | 0.86(0.06) | 0.97(0.08) | 1.13(0.09) |
|  | **SES** | 0.82(0.04)* | 0.95(0.04) | 0.91(0.06) | 1.15(0.07) | 1.11(0.09) | 0.97(0.07) |
| Continuous outcomes (Chi-Square) | **SES** | 15.11* | 29.96* | 9.05* | 1.03 | 0.08 | 0.22 |
| Categorical outcomes (OR, SE) | **Depression diagnosis** | 1.25(0.15)* | 3.21(0.33)* | 2.59(0.37)* | 2.59(0.37)* | 1.54(0.30)* | 0.60(0.11)* |
|  | **Anxiety diagnosis** | 1.11(0.20)* | 2.99(0.35)* | 2.99(0.35)* | 2.72(0.55)* | 1.27(0.37)* | 0.47(0.12)* |
|  | **PD diagnosis** | 1.23(0.61) | 2.95(0.90)* | 2.95(0.90)* | 2.62(1.55) | 2.03(1.52) | 0.78(0.41) |
|  | **ADHD diagnosis** | 2.51(0.80)* | 2.09(0.72) | 2.09(0.72) | 0.85(0.34) | 2.13(0.84) | 2.56(1.00) |
|  | **Undergraduate diploma** | 0.59(0.07)* | 0.74(0.08)* | 0.74(0.08)* | 1.26(0.18)* | 1.17(0.23)* | 0.93(0.18)* |
|  | **Entry into parenthood** | 1.29(0.21)* | 1.12(0.19)* | 1.12(0.19)* | 0.87(0.19)* | 1.04(0.29)* | 1.20(0.34)* |

Note: 1. Low ADHD, low EP (77.4%); 2. High-decreasing ADHD, low EP (8.9%); 3. Mid-decreasing ADHD, increasing EP (9.6%); 4. Increasing ADHD, increasing EP (4.2%).

Group comparation (OR, SE/Chi-square): The pairwise between-group comparisons in this section were conducted using predictors and outcomes after multiple imputation.

Baseline SES was controlled for when examining the associations between trajectory classes and outcomes.

EP, emotional problems; ADHD, attention-deficit/hyperactivity disorder; PGS, polygenic risk scores; EA, educational attainment; SES, socioeconomic Status; MDD, major depressive disorder; PD, personality disorder.

OR, odds ratio; SE, Standard Error.

* After Bonferroni correction, the p-value reached the significance level.

**Appendix S7. Joint trajectories of hyperactivity-impulsivity and emotional problems**

For the hyperactivity-impulsivity and emotional problems model, the 6-class model provided the best fit (see Table S12 and Figure S8). The 6-class model included the following categories: “Low hyperactivity-impulsivity, Low emotional problems”, “High-decreasing hyperactivity-impulsivity, Low emotional problems”, “Low hyperactivity-impulsivity, High emotional problems”, “High hyperactivity-impulsivity, Increasing emotional problems”, “Low hyperactivity-impulsivity, Increasing emotional problems” and “Increasing hyperactivity-impulsivity, Increasing emotional problems”.

**Predictors associated with the trajectories**

Results are shown in Table S14-S15 and Figure S9. Participants in the “High-decreasing hyperactivity-impulsivity, Low emotional problems” group had a higher level of ADHD PGS (M = 0.24) than those in the "Low hyperactivity-impulsivity, Low emotional problems" group (M = -0.06). Participants classified into the "High hyperactivity-impulsivity, Increasing emotional problems" group have a higher level of externalizing PGS (M = 0.42) than those in the "Low hyperactivity-impulsivity, Increasing emotional problems" group (M = -0.05).

Regarding the environmental predictors, both maternal depression and family chaos showed a general trend of being lowest in the "Low hyperactivity-impulsivity, Low emotional problems" group. SES primarily distinguished the "Low hyperactivity-impulsivity, Low emotional problems" group (M = 0.04) from the "High-decreasing hyperactivity, Low emotional problems" group (M = -0.23). In addition, numerically, maternal depression and family chaos were notably elevated in two groups characterized by higher initial ADHD or emotional symptom levels: the "High hyperactivity-impulsivity, Increasing emotional problems" group (M = 0.58 and 0.62, respectively) and the "Low hyperactivity-impulsivity, High emotional problems" group (M = 0.58 and 0.54, respectively), compared to the the other two groups (M = -0.10 and -0.10, respectively). SES was numerically lowest in the groups with higher initial symptom levels: the 'High-decreasing hyperactivity-impulsivity, Low emotional problems' group (M = -0.23), the 'Low hyperactivity-impulsivity, High emotional problems' group (M = -0.17), and the 'High hyperactivity, Increasing emotional problems' group (M = -0.44). However, these differences were not necessarily statistically significant.

**Predicting adult outcomes from joint trajectories**

Results are shown in Table S14-S15, Figure S10. Significant differences were observed in depression diagnosis and anxiety diagnosis among participants in different classes. Consistent with the overall model of emotional problems and ADHD, participants in the "Low hyperactivity-impulsivity, Low emotional problems" group showed the lowest rate of being diagnosed with depression (21.0%) or anxiety (8.8%) before 26 years old.

For sociodemographic variables beyond mental health, participants in the "Low hyperactivity-impulsivity, Low emotional problems" group had higher level of SES (M = 0.10) than participans in the “High hyperactivity-impulsivity, Increasing emotional problems” group (M = -0.96), the “Low hyperactivity-impulsivity, Increasing emotional problems” group (M = -0.28) and the “Increasing hyperactivity-impulsivity, Increasing emotional problems” group (M = -0.19). They were also significantly or non-significantly less likely to be diagnosed with depression (21.0%), anxiety (8.8%), personality disorders (1.0%), or ADHD (1.0%), were less likely to entry into parenthood (13.5%) and were more likely to earn an undergraduate degree (70.0%) than other groups before 26 years old. Conversely, the "High hyperactivity-impulsivity, Increasing emotional problems" group had the lowest SES (M = -0.96), the lowest rate of getting undergraduate diploma (36.9%), and the highest rate of having children before the age of 26 (38.7%). These results suggest that early high hyperactivity-impulsivity symptoms may be associated with lower SES, lower educational attainment, and earlier entry into parenthood.

**Appendix S8**. **Joint trajectories of inattention and emotional problems**

For the inattention and emotional problems model, the 6-class model provided the best fit. (see Table S13 and Figure S11). The 6-class model includes the following categories: "Low inattention, Low emotional problems", "High-decreasing inattention, Low emotional problems", "Low inattention, High emotional problems", "High inattention, Increasing emotional problems", "Low inattention, Increasing emotional problems" and "Increasing inattention, Increasing emotional problems".

**Predictors associated with the trajectories**

Predictors of the joint trajectories are presented in Table S16-S17 and Figure S12. The results indicated that although participants in the “Low inattention, Low emotional problem” group consistently showed lower psychiatric PGSs (for ADHD, anxiety, depression, externalizing, and MDD) and higher educational attainment PGS, the differences across trajectory groups in both psychiatric and educational attainment PGSs were not statistically significant.

In terms of environmental factors, participants in the "Low inattention, Low emotional problems" group reported the lowest levels of early maternal depression (M = -0.10) and family chaos (M = -0.10), while there were no differences in childhood SES levels among different groups. Among the groups with the highest initial symptom levels—such as the "Low inattention, High emotional problems" and "High inattention, Increasing emotional problems" groups—participants reported the highest levels of maternal depression (M = 0.48 to 0.60) and family chaos (M = 0.47 to 0.50), despite these differences not always being significant.

**Predicting adult outcomes from joint trajectories**

Outcomes of joint trajectories are shown in Table S16-S17 and Figure S13. Participants in the “Low inattention, Low emotional problems” group were less likely to be diagnosed with depression (21.0%) and anxiety (8.7%) before 16 years old, compared to the other groups. In addition, participants in the “Low inattention, low emotional problems” group, as well as in the three groups characterized by increasing emotional problems—namely the “High inattention, Increasing emotional problems,” “Low inattention, Increasing emotional problems,” and “Increasing inattention, Increasing emotional problems” groups—showed significantly or non-significantly higher rates of depression or anxiety diagnoses before the age of 26 compared with groups with low levels of emotional problems.

For variables other than mental health conditions, participants in the "Low inattention, Low emotional problems" group had higher SES levels (M = 0.11) and were more likely to obtain an undergraduate degree (70.5%) compared to most of the other groups at age 26. Participants in the "High inattention, Increasing emotional problems" group and the "Increasing inattention, Increasing emotional problems" group had the lowest SES (M = -1.04 and -0.48, respectively), although differences were not necessarily statistically significant. Additionally, participants in the "High inattention, Increasing emotional problems" group had the lowest rate of obtaining an undergraduate degree (32.0%) and the highest rate of entering parenthood at the age of 26 (29.2%), although differences were not always statistically significant. These results indicate that persistently high levels of inattention symptoms, as well as continuously increasing inattention symptoms, are associated with ongoing challenges in later mental health, academic achievement, and socioeconomic status.

**Appendix S9. The differences in the joint trajectories of ADHD subdomains and emotional problems**

The predictors and outcomes of the joint trajectories of inattention and emotional problems, as well as hyperactivity-impulsivity and emotional problems, show notable similarities. However, some predictors and outcomes function differently across the joint trajectories of ADHD subdomains and emotional problems. Specifically, ADHD PGS, externalizing PGS, and MDD PGS showed significant differences across the joint trajectories of hyperactivity-impulsivity and emotional problems, but not across the joint trajectories of inattention and emotional problems. Therefore, the group differences in ADHD PGS and externalizing PGS observed across the joint trajectories of ADHD and emotional problems were only found in the joint trajectory of hyperactivity-impulsivity and emotional problems.

Additionally, differences in childhood SES across ADHD and emotional problem joint trajectory groups primarily existed in the joint trajectory of hyperactivity-impulsivity and emotional problems, while no significant differences in childhood SES were found across the joint trajectories of inattention and emotional problems.

| **Table S12**. Relative fit indices for 1 through 5 class models for the joint trajectories of hyperactivity-impulsivity and emotional problems \| \| \| \| \|; Number of classes \| AIC \| BIC \| ABIC \| LMRT \| Entropy; 1 \| 504327 \| 504462 \| 504408 \| - \| -; 2 \| 498619 \| 498793 \| 498723 \| 0.001 \| 0.838; 3 \| 495194 \| 495407 \| 495321 \| 0.000 \| 0.818; 4 \| 493434 \| 493687 \| 493586 \| 0.004 \| 0.782; 5 \| 491782 \| 492075 \| 491957 \| 0.001 \| 0.771; **6** \| **490626** \| **490958** \| **490825** \| **0.001** \| **0.737**; 7 \| 489798 \| 490170 \| 490020 \| 0.318 \| 0.742 Note: AIC: Akaike information criterion (AIC); BIC: Bayesian information criterion; ABIC: Adjusted Bayesian information criterion; LMRT: Lo–Mendel–Rubin test. **Table S13**. Relative fit indices for 1 through 5 class models for the joint trajectories of inattention and emotional problems | | | | | |
| --- | --- | --- | --- | --- | --- |
| Number of classes | AIC | BIC | ABIC | LMRT | Entropy |
| 1 | 520618 | 520752 | 520698 | - | - |
| 2 | 516239 | 516412 | 516342 | 0.000 | 0.823 |
| 3 | 512811 | 513024 | 512938 | 0.000 | 0.791 |
| 4 | 511038 | 511291 | 511189 | 0.000 | 0.773 |
| 5 | 509888 | 510118 | 510063 | 0.000 | 0.761 |
| **6** | **508951** | **509283** | **509149** | **0.000** | **0.726** |
| 7 | 508280 | 508651 | 508502 | 0.277 | 0.729 |

Note: AIC: Akaike information criterion (AIC); BIC: Bayesian information criterion; ABIC: Adjusted Bayesian information criterion; LMRT: Lo–Mendel–Rubin test.

| **Table S14**. Descriptive statistics of predictor and outcome variables for the joint trajectory of hyperactivity-impulsivity and emotional problems based on most likely latent class membership | | | | | | | | |
| --- | --- | --- | --- | --- | --- | --- | --- | --- |
| Trajectory group (M, SD/N, %) | | | | | | |  |  |
|  |  | n | 1. Low hyper, low EP (75.4%) | 2. High-decreasing hyper, low EP (7.7%) | 3. Low hyper, high EP (4.7%) | 4. High hyper, increasing EP (2.2%) | 5. Low hyper, increasing EP (6.9%) | 6. Increasing hyper, increasing EP (3.1%) |
| Predictors(M, SD) | ADHD PGS | 12543 | -0.06(1.00) | 0.24(0.97) | 0.08(1.01) | 0.35(0.98) | 0.09(1.00) | 0.18(0.97) |
|  | Anxiety PGS | 12543 | 0.02(0.99) | -0.02(1.06) | -0.01(1.02) | -0.16(0.98) | -0.14(0.98) | -0.05(0.98) |
|  | Depression PGS | 12543 | -0.05(0.99) | 0.08(0.97) | 0.09(1.00) | 0.22(1.05) | 0.20(1.02) | 0.09(1.01) |
|  | EA PGS | 12543 | 0.04(1.00) | -0.15(0.97) | -0.19(0.94) | -0.23(1.03) | -0.03(0.97) | -0.09(0.99) |
|  | Externalizing PGS | 12543 | -0.04(1.00) | 0.20(0.97) | -0.05(1.00) | 0.42(0.95) | -0.05(1.00) | 0.22(1.03) |
|  | MDD PGS | 12543 | -0.03(1.00) | -0.01(0.98) | 0.07(1.00) | 0.28(0.97) | 0.11(1.00) | 0.08(1.05) |
|  | Maternal depression | 10692 | -0.10(0.96) | 0.28(1.01) | 0.58(1.11) | 0.58(1.00) | 0.21(1.02) | 0.22(1.08) |
|  | Family chaos | 10676 | ^‘^-0.10(0.98) | 0.41(0.92) | 0.54(1.02) | 0.62(1.00) | 0.13(0.98) | 0.19(0.98) |
|  | SES | 18769 | 0.04(0.99) | -0.23(0.96) | -0.17(1.02) | -0.44(1.00) | 0.05(0.98) | -0.09(1.06) |
| Continuous outcomes(M, SD) | SES | 7999 | 0.10(0.93) | -0.20(1.12) | -0.20(1.07) | -0.96(1.44) | -0.28(1.08) | -0.19(1.15) |
|  | Depression diagnosis | 7790 | 1209(21.0%) | 155(28.5%) | 93(30.7%) | 49(41.2%) | 407(51.9%) | 104(37.8%) |
|  | Anxiety diagnosis | 7790 | 506(8.8%) | 55(10.1%) | 43(14.2%) | 18(15.1%) | 186(23.7%) | 33(12.0%) |
|  | PD diagnosis | 7783 | 57(1.0%) | 7(1.3%) | 5(1.7%) | 3(2.6%) | 27(3.5%) | 6(2.2%) |
| Categorical outcomes(N, %) | ADHD diagnosis | 7783 | 59(1.0%) | 22(4.1%) | 5(1.7%) | 17(14.7%) | 20(2.6%) | 12(4.4%) |
|  | Undergraduate diploma | 7845 | 4067(70.0%) | 278(51.7%) | 191(60.3%) | 41(36.9%) | 488(61.1%) | 157(57.7%) |
|  | Entry into parenthood | 8347 | 827(13.5%) | 139(22.9%) | 62(18.5%) | 53(38.7%) | 128(15.1%) | 70(22.6%) |
|  |  |  |  |  |  |  |  |  |

Note: All predictors and outcomes have been standardized to have a mean of zero and SD of 1.

EP, emotional problems; hyper, hyperactivity-impulsivity; ADHD, attention-deficit/hyperactivity disorder; PGS, polygenic risk scores; EA, educational attainment; SES, socioeconomic Status; MDD, major depressive disorder; PD, personality disorder.

M, mean; SD, standard deviation; OR, odds ratio.

Trajectory group (M, SD): The mean and standard deviation of predictors in each trajectory group based on the most likely latent class membership.

Trajectory group(N, %): The number and proportion of participants in each trajectory group.

*Descriptive statistics based on “most likely classes” may be inconsistent with the results of subsequent pairwise comparisons using the three-step method, as the latter accounts for uncertainty in class membership.

| **Table S15**. Pairwise comparisons of predictor and outcome variables for the joint trajectory of hyperactivity-impulsivity and emotional problems across classes in the three-step method | | | | | | | | | | | | | | | | |
| --- | --- | --- | --- | --- | --- | --- | --- | --- | --- | --- | --- | --- | --- | --- | --- | --- |
| Trajectory group (OR, SE) | | | | | | | | | | | | | | | | |
|  |  | 2 VS. 1 | 3 VS. 1 | 4 VS. 1 | 5 vs. 1 | 6 vs. 1 | 3 VS. 2 | 4 VS. 2 | 5 VS. 2 | 6 vs. 2 | 4 vs. 3 | 5 VS. 3 | 6 vs. 3 | 5 vs. 4 | 6 VS. 4 | 6 vs. 5 |
| Predictors | **ADHD PGS** | 1.61(0.14)* | 1.37(0.17) | 1.47(0.21) | 1.25(0.10) | 1.24(0.14) | 0.85(0.12) | 0.91(0.15) | 0.77(0.08) | 0.77(0.11) | 1.08(0.20) | 0.91(0.13) | 0.91(0.15) | 0.85(0.13) | 0.84(0.16) | 1.00(0.13) |
| (OR, SE) | Anxiety PGS | 1.17(0.12) | 0.97(0.12) | 0.84(0.12) | 0.94(0.07) | 1.08(0.13) | 0.83(0.12) | 0.72(0.13) | 0.80(0.09) | 0.92(0.15) | 0.87(0.16) | 0.97(0.13) | 1.11(0.19） | 1.12(0.17) | 1.28(0.25) | 1.15(0.16) |
|  | Depression PGS | 1.09(0.11) | 0.95(0.13) | 0.93(0.17) | 1.21(0.12) | 1.06(0.16) | 0.88(0.14) | 0.85(0.18) | 1.11(0.14) | 0.98(0.17) | 0.97(0.21) | 1.27(0.20) | 1.11(0.21) | 1.30(0.25) | 1.15(0.27) | 0.88(0.15) |
|  | EA PGS | 0.97(0.09) | 0.74(0.09) | 0.94(0.18) | 0.98(0.08) | 1.05(0.11) | 0.77(0.11) | 0.97(0.21) | 1.01(0.11) | 1.08(0.15) | 1.26(0.28) | 1.32(0.19) | 1.41(0.22) | 1.04(0.21) | 1.12(0.25) | 1.07(0.13) |
|  | **Externalizing PGS** | 1.11(0.10) | 0.98(0.11) | 1.37(0.17) | 0.91(0.08) | 1.39(0.17) | 0.88(0.12) | 1.23(0.18) | 0.82(0.09) | 1.25(0.18) | 1.39(0.22) | 0.93(0.13) | 1.42(0.23) | 0.67(0.09)* | 1.02(0.17) | 1.53(0.21) |
|  | **MDD PGS** | 0.78(0.08) | 1.05(0.14) | 1.56(0.26) | 1.02(0.09) | 0.89(0.12) | 1.34(0.20) | 1.99(0.37) | 1.30(0.15) | 1.13(0.18) | 1.49(0.30) | 0.98(0.15) | 0.85(0.15) | 0.65(0.12)* | 0.57(0.12)* | 0.87(0.13) |
|  | **Maternal depression** | 1.37(0.12)* | 1.94(0.20)* | 1.78(0.24)* | 1.55(0.12)* | 1.46(0.17) | 1.42(0.17) | 1.30(0.20) | 1.13(0.11) | 1.06(0.15) | 0.92(0.14) | 0.80(0.09) | 0.75(0.11) | 0.87(0.12) | 0.82(0.15) | 0.94(0.12) |
|  | **Family chaos** | 1.82(0.16)* | 1.92(0.27)* | 1.78(0.28) | 1.29(0.12) | 1.34(0.16) | 1.06(0.16) | 0.98(0.17) | 0.71(0.08)* | 0.74(0.10) | 0.93(0.18) | 0.67(0.11)* | 0.70(0.12) | 0.73(0.12) | 0.76(0.15) | 1.04(0.14) |
|  | **SES** | 0.78(0.07)* | 1.93(0.14) | 0.70(0.11) | 0.95(0.08) | 0.98(0.13) | 1.20(0.20) | 0.89(0.15) | 1.22(0.13) | 1.26(0.19) | 0.75(0.16) | 1.02(0.18) | 1.06(0.20) | 1.36(0.22) | 1.42(0.29) | 1.04(0.15) |
| Continuous  outcomes  (Chi-Square) | **SES** | 6.03 | 5.87 | 35.55* | 79.75* | 12.02* | 0.18 | 22.41* | 14.26* | 0.84 | 19.42* | 5.83 | 0.16 | 12.34* | 17.96* | 5.73 |
|  | **Depression diagnosis** | 2.03(0.33)* | 1.77(0.48) | 4.08(1.12)* | 7.50(0.95)* | 3.33(0.57)* | 0.87(0.26)* | 2.01(0.63)* | 3.69(0.63)* | 1.64(0.35)* | 2.30(0.86) | 4.23(1.21)* | 1.88(0.57)* | 1.84(0.52)* | 0.82(0.26)* | 0.44(0.08)* |
|  | **Anxiety diagnosis** | 1.54(0.39)* | 2.05(0.72) | 3.07(1.09) | 5.48(0.87)* | 1.94(0.50)* | 1.33(0.54) | 1.99(0.85) | 3.55(0.86)* | 1.25(0.42)* | 1.50(0.72) | 2.67(0.96) | 0.94(0.38) | 1.78(0.63) | 0.63(0.27) | 0.35(0.09)* |
|  | PD diagnosis | 1.35(1.29) | 1.92(2.40) | 6.50(5.16) | 7.08(3.73) | 4.36(2.82) | 1.42(2.08) | 4.80(5.42) | 5.23(4.35) | 3.23(3.17) | 3.40(4.63) | 3.69(4.47) | 2.27(2.88) | 1.09(0.78) | 0.67(0.58) | 0.62(0.33) |
| Categorical outcomes | ADHD diagnosis | 8.07(4.54) | 2.46(2.81) | 51.60(27.36) | 5.35(3.06) | 7.30(4.62) | 0.30(0.35) | 6.39(3.05) | 0.66(0.27) | 0.91(0.48) | 21.00(24.08) | 2.18(2.51) | 2.97(3.45) | 0.10(0.04) | 0.14(0.08) | 1.36(0.67) |
| (OR, SE) | **Undergraduate diploma** | 0.46(0.07)* | 0.72(0.17)* | 0.23(0.09) | 0.56(0.07)* | 0.56(0.10)* | 1.56(0.44)* | 0.50(0.21) | 1.22(0.22)* | 1.21(0.28)* | 0.32(0.14) | 0.78(0.21)* | 0.78(0.23)* | 2.43(0.93) | 2.41(1.01) | 0.99(0.20)* |
|  | **Entry into parenthood** | 1.63(0.28)* | 1.36(0.35)* | 3.44(0.98)* | 1.18(0.19)* | 1.68(0.33)* | 0.84(0.25)* | 2.11(0.70)* | 0.73(0.14)* | 1.03(0.25)* | 2.52(0.97) | 0.87(0.26)* | 1.23(0.39)* | 0.34(0.11)* | 0.49(0.17) | 1.42(0.32)* |
|  |  |  |  |  |  |  |  |  |  |  |  |  |  |  |  |  |

Note: 1. Low hyperactivity-impulsivity, Low emotional problems (75.4%); 2. High-decreasing hyperactivity-impulsivity, Low emotional problems (7.3%); 3. Low hyperactivity-impulsivity, High emotional problems (4.7%); 4. High hyperactivity-impulsivity, Increasing emotional problems (2.2%); 5. Low hyperactivity-impulsivity, Increasing emotional problems (6.9%); 6. Increasing hyperactivity-impulsivity, Increasing emotional problems (3.1%).

Baseline SES was controlled for when examining the associations between trajectory classes and outcomes.

EP, emotional problems; ADHD, attention-deficit/hyperactivity disorder; PGS, polygenic risk scores; EA, educational attainment; SES, socioeconomic Status; MDD, major depressive disorder; PD, personality disorder.

OR, odds ratio; SE, Standard Error.

* After Bonferroni correction, the p-value reached the significance level.

| **Table S16**. Descriptive statistics of predictor and outcome variables for the joint trajectory of inattention and emotional problems based on most likely latent class membership | | | | | | | | |
| --- | --- | --- | --- | --- | --- | --- | --- | --- |
| Trajectory group (M, SD/N, %) | | | | | | |  |  |
|  |  | n | 1.Low inatt, low EP (75.6%) | 2. High-decreasing inatt, low EP (6.9%) | 3. Low inatt, high EP (4.8%) | 4. High inatt, increasing EP (2.4%) | 5. Low inatt, increasing EP (6.6%) | 6. Increasing inatt, increasing EP (3.8%) |
| Predictors(M, SD) | ADHD PGS | 12543 | -0.05(1.00) | 0.18(0.99) | 0.05(0.98) | 0.35(0.96) | 0.11(0.99) | 0.18(0.96) |
|  | Anxiety PGS | 12543 | 0.03(1.00) | -0.06(1.01) | -0.03(1.01) | -0.16(1.02) | -0.11(1.00) | -0.05(1.00) |
|  | Depression PGS | 12543 | -0.04(1.00) | 0.03(0.98) | 0.10(0.96) | 0.25(1.04) | 0.19(1.02) | 0.08(0.99) |
|  | EA PGS | 12543 | 0.03(1.00) | -0.09(1.02) | -0.14(0.95) | -0.22(0.97) | -0.04(0.96) | -0.06(1.04) |
|  | Externalizing PGS | 12539 | -0.03(1.00) | 0.13(1.01) | -0.10(0.98) | 0.32(1.00) | -0.03(1.01) | 0.25(0.99) |
|  | MDD PGS | 12543 | -0.03(1.00) | 0.04(0.98) | 0.05(1.02) | 0.22(0.95) | 0.12(1.03) | 0.02(0.98) |
|  | Maternal depression | 10692 | -0.10(0.96) | 0.26(1.06) | 0.60(1.08) | 0.48(1.01) | 0.24(1.02) | 0.22(1.06) |
|  | Family chaos | 10676 | -0.10(0.98) | 0.29(0.97) | 0.50(1.00) | 0.47(1.04) | 0.17(0.98) | 0.32(0.98) |
|  | SES | 18769 | 0.02(1.00) | -0.05(0.96) | -0.20(1.01) | -0.26(1.08) | 0.03(1.00) | 0.03(1.03) |
| Continuous outcomes(M, SD) | SES | 7999 | 0.11(0.94) | -0.22(1.03) | -0.14(1.07) | -1.04(1.22) | -0.27(1.08) | -0.48(1.14) |
|  | Depression diagnosis | 7790 | 1227(21.0%) | 129(27.0%) | 115(33.2%) | 59(46.1%) | 376(50.0%) | 111(44.4%) |
|  | Anxiety diagnosis | 7790 | 506(8.7%) | 39(8.2%) | 49(14.2%) | 19(14.8%) | 188(25.0%) | 40(16.0%) |
|  | PD diagnosis | 7783 | 59(1.0%) | 5(1.0%) | 9(2.6%) | 2(1.6%) | 23(3.1%) | 7(2.8%) |
| Categorical outcomes(N, %) | ADHD diagnosis | 7783 | 60(1.0%) | 13(2.7%) | 8(2.3%) | 15(12.2%) | 20(2.7%) | 19(7.6%) |
|  | Undergraduate diploma | 7846 | 4136(70.5%) | 246(51.0%) | 217(60.6%) | 39(32.0%) | 468(60.9%) | 116(46.4%) |
|  | Entry into parenthood | 8347 | 869(14.0%) | 99(18.9%) | 68(18.0%) | 42(29.2%) | 133(16.3%) | 68(23.0%) |
|  |  |  |  |  |  |  |  |  |

Note: All predictors and outcomes have been standardized to have a mean of zero and SD of 1.

EP, emotional problems; inatt, inattention; ADHD, attention-deficit/hyperactivity disorder; PGS, polygenic risk scores; EA, educational attainment; SES, socioeconomic Status; MDD, major depressive disorder; PD, personality disorder.

M, mean; SD, standard deviation; OR, odds ratio.

Trajectory group (M, SD): The mean and standard deviation of predictors in each trajectory group based on the most likely latent class membership.

Trajectory group(N, %): The number and proportion of participants in each trajectory group.

*Descriptive statistics based on “most likely classes” may be inconsistent with the results of subsequent pairwise comparisons using the three-step method, as the latter accounts for uncertainty in class membership.

| **Table S17**. Pairwise comparisons of predictor and outcome variables for the joint trajectory of inattention and emotional problems across classes in the three-step method | | | | | | | | | | | | | | | | |
| --- | --- | --- | --- | --- | --- | --- | --- | --- | --- | --- | --- | --- | --- | --- | --- | --- |
|  |  | 2 VS. 1 | 3 VS. 1 | 4 VS. 1 | 5 vs. 1 | 6 vs. 1 | 3 VS. 2 | 4 VS. 2 | 5 VS. 2 | 6 vs. 2 | 4 vs. 3 | 5 VS. 3 | 6 vs. 3 | 5 vs. 4 | 6 VS. 4 | 6 vs. 5 |
| Predictors(OR, SE) | ADHD PGS | 1.29(0.10) | 1.25(0.15) | 1.56(0.24) | 1.28(0.10) | 1.34(0.16) | 0.97(0.13) | 1.21(0.21) | 0.99(0.10) | 1.04(0.14) | 1.25(0.23) | 1.03(0.14) | 1.07(0.17) | 0.82(0.13) | 0.86(0.17) | 1.05(0.15) |
|  | Anxiety PGS | 1.02(0.08) | 0.91(0.11) | 0.75(0.12) | 0.96(0.07) | 1.02(0.12) | 0.89(0.12) | 0.73(0.13) | 0.94(0.09) | 1.00(0.14) | 0.82(0.16) | 1.05(0.15) | 1.12(0.18) | 1.28(0.22) | 1.37(0.28) | 1.07(0.14) |
|  | Depression PGS | 1.00(0.10) | 1.00(0.13) | 0.93(0.18) | 1.25(0.13) | 1.18(0.17) | 0.99(0.15) | 0.93(0.20) | 1.24(0.15) | 1.18(0.20) | 0.93(0.21) | 1.25(0.20) | 1.19(0.22) | 1.34(0.28) | 1.28(0.31) | 0.95(0.16) |
|  | EA PGS | 0.94(0.09) | 0.83(0.10) | 0.74(0.13) | 1.04(0.08) | 0.98(0.12) | 0.89(0.12) | 0.79(0.16) | 1.11(0.12) | 1.05(0.15) | 0.89(0.18) | 1.25(0.17) | 1.18(0.19) | 1.41(0.27) | 1.34(0.30) | 0.94(0.13) |
|  | Externalizing PGS | 1.11(0.09) | 0.86(0.10) | 1.00(0.16) | 0.93(0.09) | 1.20(0.12) | 0.78(0.10) | 0.90(0.17) | 0.84(0.09) | 1.08(0.14) | 1.16(0.22) | 1.08(0.15) | 1.39(0.20) | 0.93(0.17) | 1.20(0.23) | 1.30(0.16) |
|  | MDD PGS | 1.01(0.10) | 1.02(0.14) | 1.16(0.20) | 0.68(0.10) | 0.86(0.11) | 1.02(0.16) | 1.16(0.23) | 0.95(0.11)\ | 0.86(0.13) | 1.13(0.25) | 0.94(0.15) | 0.84(0.15) | 0.83(0.16) | 0.74(0.16) | 0.90(0.13) |
|  | **Maternal depression** | 1.45(0.12)* | 2.08(0.20)* | 2.06(0.28)* | 1.58(0.13)* | 1.44(0.17) | 1.43(0.16) | 1.42(0.22) | 1.09(0.11) | 0.99(0.14) | 0.99(0.15) | 0.76(0.08) | 0.69(0.10)* | 0.77(0.11) | 0.70(0.13) | 0.91(0.12) |
|  | **Family chaos** | 1.51(0.13)* | 1.92(0.23)* | 1.42(0.27) | 1.30(0.12) | 1.69(0.21)* | 1.28(0.17) | 0.94(0.20) | 0.87(0.09) | 1.12(0.15) | 0.74(0.16) | 0.68(0.10)* | 0.88(0.14) | 0.92(0.18) | 1.19(0.27) | 1.29(0.18) |
|  | SES | 0.92(0.08) | 0.82(0.12) | 1.04(0.20) | 0.96(0.08) | 1.10(0.13) | 0.89(0.14) | 1.13(0.24) | 1.05(0.11) | 1.20(0.16) | 1.27(0.30) | 1.18(0.19) | 1.35(0.23) | 0.93(0.19) | 1.06(0.25) | 1.14(0.16) |
| Continuous outcomes (Chi-Square) | **SES** | 29.51* | 4.54 | 63.12* | 79.23* | 50.82* | 2.63 | 25.28* | 2.57 | 12.35* | 35.14* | 8.27 | 19.77* | 21.46* | 4.47 | 7.12 |
|  | **Depression diagnosis** | 1.79(0.30)* | 2.25(0.48)* | 4.96(1.36)* | 6.75(0.86)* | 5.62(1.19)* | 1.26(0.32)* | 2.78(0.90)* | 3.78(0.68)* | 3.15(0.81)* | 2.21(0.75)* | 3.00(0.71)* | 2.50(0.71)* | 1.36(0.39)* | 1.13(0.39)* | 0.83(0.19)* |
|  | **Anxiety diagnosis** | 1.09(0.34)* | 2.07(0.64)* | 3.24(1.06)* | 6.16(1.00)* | 3.33(0.94)* | 1.91(0.79) | 2.98(1.36) | 5.67(1.71)* | 3.06(1.22) | 1.57(0.68) | 2.98(0.96)* | 1.61(0.62) | 1.90(0.63)* | 1.03(0.43) | 0.54(0.15)* |
|  | PD diagnosis | 1.08(1.12) | 3.86(2.68) | 3.20(3.18) | 5.89(2.89) | 6.37(3.89) | 3.58(4.07) | 2.98(4.28) | 5.47(5.41) | 5.91(6.48) | 0.83(0.92) | 1.53(1.00) | 1.65(1.24) | 1.84(1.77) | 1.99(2.16) | 1.08(0.59) |
| Categorical outcomes(OR, SE) | ADHD diagnosis | 5.02(3.52) | 5.47(3.99) | 41.27(23.13) | 6.26(3.77) | 21.53(13.14) | 1.09(0.81) | 8.23(5.16) | 1.25(0.66) | 4.29(2.53) | 7.55(5.17) | 1.14(0.77) | 3.94(2.59) | 0.15(0.07) | 0.52(0.26) | 3.44(1.49) |
|  | **Undergraduate diploma** | 0.30(0.05)* | 0.67(0.15)* | 0.12(0.04)* | 0.52(0.07)* | 0.17(0.05)* | 2.22(0.58)* | 0.40(0.16) | 1.72(0.32)* | 0.56(0.17)* | 0.18(0.07) | 0.78(0.19)* | 0.25(0.08)* | 4.36(1.60) | 1.42(0.63) | 0.32(0.09)* |
|  | **Entry into parenthood** | 1.37(0.26)* | 1.29(0.32)* | 2.06(0.61)* | 1.23(0.20)* | 2.08(0.51)* | 0.94(0.28)* | 1.51(0.54) | 0.90(0.19)* | 1.52(0.45)* | 1.60(0.60) | 0.95(0.27)* | 1.61(0.54)* | 0.60(0.19)* | 1.01(0.39) | 1.70(0.47)* |
|  |  |  |  |  |  |  |  |  |  |  |  |  |  |  |  |  |

Note: 1. Low inattention, Low emotional problems (75.6%); 2. High-decreasing inattention, Low emotional problems (6.9%); 3. Low inattention, High emotional problems (4.8%); 4. High inattention, Increasing emotional problems (2.4%); 5. Low inattention, Increasing emotional problems (6.6%); 6. Increasing inattention, Increasing emotional problems (3.8%).

Baseline SES was controlled for when examining the associations between trajectory classes and outcomes.

EP, emotional problems; ADHD, attention-deficit/hyperactivity disorder; PGS, polygenic risk scores; EA, educational attainment; SES, socioeconomic Status; MDD, major depressive disorder; PD, personality disorder;

OR, odds ratio; SE, Standard Error.

* After Bonferroni correction, the p-value reached the significance level.

An odds ratio (OR) greater than 1.00 in the 2 vs. 1 comparison column was associated with higher odds of class membership in group 2 (comparison group) compared to group 1 (reference group). For example, a higher PGS for ADHD was associated with greater odds of class membership in group 2 compared to group 1.

ADHD

|  | class1 | class2 | class3 | class4 |
| --- | --- | --- | --- | --- |
| age4 | 7.86 | 38.1 | 23.56 | 14.59 |
| age7 | 6.76 | 37.65 | 21.04 | 15.89 |
| age9 | 5.84 | 34.91 | 18.05 | 18.68 |
| age12 | 4.56 | 29.7 | 13.3 | 19.05 |
| age21 | 4.66 | 19.1 | 7.99 | 26.61 |

**Figure S1**. Trajectories of ADHD

Note: class 1, Low ADHD (13484, 80.0%; S = -0.24, I = 7.61); class 2: High ADHD (536, 3.2%; S = -1.58, I=41.49); class 3: Decreasing ADHD (2044, 12.1%; S = -1.31, I = 24.09); class 4: Increasing ADHD (790, 4.7%; S = 0.88 , I = 14.77).

Hyperactivity-impulsivity

|  | class1 | class2 | class3 | class4 |
| --- | --- | --- | --- | --- |
| age4 | 4.18 | 19.23 | 13.29 | 6.96 |
| age7 | 2.85 | 18.83 | 11.02 | 6.28 |
| age9 | 2.24 | 18.57 | 9.7 | 6.86 |
| age12 | 1.62 | 16.26 | 6.49 | 6.81 |
| age21 | 1.52 | 15.94 | 3.41 | 11.99 |

**Figure S2**. Trajectories of hyperactivity-impulsivity

Note: class 1, Low hyperactivity-impulsivity (13688, 81.2%; S = -0.18, I = 3.62); class 2: High hyperactivity-impulsivity (435, 2.5%; S = -0.36, I = 19.90); class 3: Decreasing hyperactivity-impulsivity (1992, 11.8%; S = -0.83, I = 13.33); class 4: Increasing hyperactivity-impulsivity (733, 4.4%; S = 0.43, I = 6.15).

Inattention

|  | class1 | class2 | class3 | class4 | class5 | class6 | class7 | class8 |
| --- | --- | --- | --- | --- | --- | --- | --- | --- |
| age8 | 2.7 | 9.79 | 9.78 | 5.02 | 12.89 | 19.3 | 21.89 | 4.42 |
| age12 | 2.93 | 16.31 | 8.86 | 8.49 | 13.28 | 15.16 | 21.27 | 6.1 |
| age14 | 2.47 | 17.67 | 7.56 | 9.95 | 13.28 | 12.38 | 21.05 | 6.59 |
| age16 | 2 | 19.25 | 5.38 | 9.88 | 12.17 | 9.34 | 18.86 | 6.2 |
| age21 | 1.76 | 23.47 | 3.09 | 16.23 | 9.88 | 3.99 | 15.5 | 8.25 |

**Figure S3**. Trajectories of inattention

Note: class 1, Low inattention (10564, 62.7%, S = -0.08, I = 2.85); class 2, High-increasing inattention (219, 1.3%, S = 1.02, I = 10.64); class 3, Mid-decreasing inattention (2508, 14.9%, S = -0.51, I = 9.72); class 4, Low-sharp increasing inattention (428, 2.5%, S = 0.84, I = 4.79); class 5, Mid inattention (728, 4.3%, S = -0.24, I = 13.00); class 6, High-decreasing inattention (492, 2.9%, S = -1.18, I = 19.29); class 7, High inattention (332, 2.0%, S = -0.47, I = 22.15); class 8, Low-mild increasing (1583, 9.4%, S = 0.27, I = 4.47).

Emotional problems

|  | class1 | class2 | class3 | class4 |
| --- | --- | --- | --- | --- |
| age4 | 0.94 | 4.76 | 3.78 | 1.29 |
| age7 | 1.59 | 6.17 | 4.06 | 3.07 |
| age9 | 1.03 | 6.31 | 3.45 | 3.18 |
| age12 | 1.1 | 6.38 | 2.95 | 3.66 |
| age21 | 1.44 | 6.76 | 2.22 | 6.11 |

**Figure S4**. Trajectories of emotional problems

Note: class 1, Low emotional problems (14689, 75.3%, S = 0.025, I = 1.02); class 2, High emotional problems (640, 3.3%, S = 0.13, I = 4.91); class 3, Decreasing emotional problems (1992, 10.2%, S = -0.08, I = 3.63); class 4, Increasing emotional problems (2197, 11.3%, S = 0.26, I = 1.48).

**Figure S5**. Joint trajectories of ADHD and emotional problems by using subsample with inverse probability weighting

Note: class 1, Low ADHD, Low emotional problems (9767, 77.4%, S_ADHD_ = -0.30, I_ADHD_ = 8.55, S_EP_ = 0.01, I_EP_ = 1.47); class 2, High-decreasing ADHD, Low emotional problems (1212, 9.6%, S_ADHD_ = -1.68, I_ADHD_ = 31.42, S_EP_ = 0.05, I_EP_ = 2.15); class 3, Low ADHD, Increasing emotional problems (1120, 8.9%, S_ADHD_ = -0.14, I_ADHD_ = 10.33, S_EP_ = 0.27, I_EP_ = 1.98); class 4, Increasing ADHD, Increasing emotional problems (526, 4.2%, S_ADHD_ = 1.03, I_ADHD_ = 16.94, S_EP_ = 0.15, I_EP_ = 1.60).

**Figure S6**. Predictors for the joint trajectory of ADHD and emotional problems based on the most likely latent class membership by using subsample with inverse probability weighting

Note: All predictors have been standardized to have a mean of zero and SD of 1. The error bars represent the standard error of the data.

EP, emotional problems; ADHD, attention-deficit/hyperactivity disorder; Hyper, hyperactivity-impulsivity; PGS, polygenic risk scores; EA, educational attainment; SES, socioeconomic Status; MDD, major depressive disorder; PD, personality disorder.

**Figure S7**. Categorical outcomes of the joint trajectory of ADHD and emotional problems based on most likely latent class membership by using subsample with inverse probability weighting

Note: EP, emotional problems; ADHD, attention-deficit/hyperactivity disorder; PD diagnosis, personality disorder diagnosis.

**Class 1, Low hyperactivity-impulsivity, low emotional problems**

**Class 2, High-decreasing hyperactivity-impulsivity, low emotional problems**

**Class 3, Low hyperactivity-impulsivity, high emotional problems**

**Class 4, High hyperactivity-impulsivity, increasing emotional problems**

**Class 5, Low hyperactivity-impulsivity, increasing emotional problems**

**Class 6, Increasing hyperactivity-impulsivity, increasing emotional problems**

**Figure S8**. Joint trajectories of hyperactivity-impulsivity and emotional problems

Note: class 1, low hyperactivity-impulsivity, low emotional problems (15141, 75.4%, S_hyper_ = -0.178, I_hyper_ = 3.604, S_EP_ = 0.021, I_EP_ = 1.228); class 2, high-decreasing hyperactivity-impulsivity, low emotional problems (1551, 7.3%, S_hyper_ = -0.91, I_hyper_ = 14.28, S_EP_ = 0.05, I_EP_ = 1.68); class 3, low hyperactivity-impulsivity, high emotional problems (934, 4.7%, S_hyper_ = -0.362, I_hyper_ = 6.849, S_EP_ = -0.087, I_EP_ = 4.893); class 4, high hyperactivity-impulsivity, increasing emotional problems (446, 2.2%, S_hyper_ = -0.33, I_hyper_ = 19.43, S_EP_ = 0.17, I_EP_ = 2.67); class 5, low hyperactivity-impulsivity, increasing emotional problems (1382, 6.9%, S_hyper_ = -0.17, I_hyper_ = 4.57, S_EP_ = 0.28, I_EP_ = 1.72); class 6, increasing hyperactivity-impulsivity, increasing emotional problems (618, 3.1%, S_hyper_ = 0.52, I_hyper_ = 5.89, S_EP_ = 0.11, I_EP_ = 1.61).

**Figure S9**. Predictors for the joint trajectory of hyperactivity-impulsivity and emotional problems based on most likely latent class membership

Note: All predictors have been standardized to have a mean of zero and SD of 1. The error bars represent the standard error of the data.

EP, emotional problems; ADHD, attention-deficit/hyperactivity disorder; Hyper, hyperactivity-impulsivity; PGS, polygenic risk scores; EA, educational attainment; SES, socioeconomic Status; MDD, major depressive disorder; PD, personality disorder.

**Figure S10**. Categorical adult outcomes of the joint trajectory of ADHD and emotional problems based on most likely class membership

Note: All predictors have been standardized to have a mean of zero and SD of 1. The error bars represent the standard error of the data.

EP, emotional problems; ADHD, attention-deficit/hyperactivity disorder; Hyper, hyperactivity-impulsivity; PD diagnosis, personality disorder diagnosis.

**Class 1, Low inattention, Low emotional problems**

**Class 2, High-decreasing inattention, Low emotional problems**

**Class 3, Low inattention, High emotional problems**

**Class 4, High inattention, Increasing emotional problems**

**Class 5, Low inattention, Increasing emotional problems**

**Class 6, Increasing inattention, Increasing emotional problems**

**Figure S11**. Joint trajectories of inattention and emotional problems

Note: class 1, Low inattention, Low emotional problems (15176, 75.6%, S_inattention_ = -0.08, I_inattention_ = 3.78, S_EP_ = 0.02, I_EP_ = 1.22); class 2, High-decreasing inattention, Low emotional problems (1376, 6.9%, S_inattention_ = -0.80, I_inattention_ = 14.87, S_EP_ = 0.03, I_EP_ = 1.71); class 3, Low inattention, High emotional problems (958, 4.8%, S_inattention_ = -0.21, I_inattention_ = 6.68, S_EP_ = -0.07, I_EP_ = 4.89); class 4, High inattention, Increasing emotional problems (475, 2.4%, S_inattention_ = -0.37, I_inattention_ = 20.64, S_EP_ = 0.18, I_EP_ = 2.59); class 5, Low inattention, Increasing emotional problems (1321, 6.6%, S_inattention_ = -0.025, I_inattention_ = 5.14 S_EP_ = 0.27, I_EP_ = 1.77); class 6, Increasing inattention, Increasing emotional problems (766, 3.8%, S_inattention_ = 0.79, I_inattention_ = 6.69, S_EP_ = 0.15, I_EP_ = 1.46).

**Figure S12**. Predictors for the joint trajectory of inattention and emotioanl problems

Note: All predictors have been standardized to have a mean of zero and SD of 1. The error bars represent the standard error of the data.

EP, emotional problems; ADHD, attention-deficit/hyperactivity disorder; Inatt, inattention; PGS, polygenic risk scores; EA, educational attainment; SES, socioeconomic Status; MDD, major depressive disorder; PD, personality disorder.

**Figure S13**. Categorical adult outcomes of the joint trajectory of inattention and emotional problems based on most likely class membership

Note: All predictors have been standardized to have a mean of zero and SD of 1. The error bars represent the standard error of the data.

EP, emotional problems; ADHD, attention-deficit/hyperactivity disorder; Inatt, inattention; PD diagnosis, personality disorder diagnosis.

Conners, Conners inattention symptoms at age 26; SDQ_EP, SDQ emotional problems at age 26; SDQ_hyper, SDQ hyperactivity symptoms at age 26; SES, Socioeconomic status at age 26.
